# The Mechanisms Underlying the Relationship Between Self‐Compassion and Psychological Outcomes in Adult Populations: A Systematic Review

**DOI:** 10.1002/smi.70090

**Published:** 2025-07-28

**Authors:** Jun Wang, Constance H. C. Drossaert, Maleah Knevel, Liyan Chen, Ernst T. Bohlmeijer, Maya J. Schroevers

**Affiliations:** ^1^ Department of Health Sciences University Medical Center Groningen University of Groningen Groningen the Netherlands; ^2^ Department of Psychology Health and Technology University of Twente Enschede the Netherlands

**Keywords:** mechanism, mediation, psychological health, self‐compassion

## Abstract

Self‐compassion has emerged as a protective factor for psychological health and well‐being. Interest has grown in understanding the mechanisms that explain how self‐compassion contributes to improved psychological outcomes. This systematic review aimed to identify and synthesise the results of studies that investigated the mediators underlying the relationship between self‐compassion and psychological outcomes. Potential eligible studies were searched on Embase, APA PsycINFO, PubMed, and Web of Science (search up till January 2025). Included were peer‐reviewed, English‐language studies investigating mediators between self‐compassion and psychological outcomes. Study quality was assessed using the Mediation Quality Checklist. A total of 113 studies were included, and most were cross‐sectional and focused on psychological symptoms rather than well‐being. Repetitive negative thinking, such as worry and rumination, was the most studied process and found to be significantly mediating self‐compassion and reduced psychological symptoms across studies. There is also growing evidence for experiential avoidance and mindfulness as significant mediators. Limited studies precluded drawing conclusions about other coping strategies, self‐concept, and social factors. This systematic review points toward a significant mediational role of maladaptive (e.g. repetitive negative thinking, experiential avoidance) and adaptive (e.g. mindfulness) emotion regulation and coping strategies, that explain the association of self‐compassion with psychological outcomes. Future studies with more rigorous designs including intensive longitudinal and experimental designs are needed to clarify causality.

## 1 Introduction

Interest in self‐compassion has increased over the past decade, in research, society, and health care. *Self‐compassion* refers to being aware of and emotionally touched by suffering, understanding its universality, combined with the intention to act or acting to ease this suffering (Gilbert 2020; Neff 2003a; Strauss et al. 2016). A recent meta‐analysis encompassing 168 studies revealed that higher levels of self‐compassion are moderately related to lower levels of psychological distress and higher levels of well‐being (Chio et al. 2021). These results are corroborated by a systematic review of 16 studies in people with chronic physical illness (Hughes et al. 2021). Given these psychological benefits of self‐compassion, interventions have been developed that aim to cultivate self‐compassion, such as Compassion‐Focused Therapy (CFT; Gilbert 2009), Mindful Self‐Compassion (MSC; Germer and Neff 2019), Compassion Cultivation Training (CCT; Jazaieri et al. 2014), and Mindfulness‐Based Compassionate Living (MBCL; Bartels‐Velthuis et al. 2016). These interventions have been found to be effective in reducing psychological symptoms and improving well‐being (Austin et al. 2021; Kilic et al. 2021; Kirby 2017; Luo et al. 2021; Mistretta and Davis 2022; Petrocchi et al. 2024). To achieve a clear understanding of the potential benefits of self‐compassion and such interventions, it is crucial to identify potential pathways that explain these beneficial outcomes. The aim of this systematic review is to synthesise the current empirical evidence concerning the mediating factors underlying the association of self‐compassion with psychological symptoms and well‐being. Gaining these insights into mechanisms can facilitate the integration of theory, research, and practice of self‐compassion to advance well‐being and health (Kazdin 2007). Moreover, this knowledge could guide future clinical trials by identifying potential mechanisms to investigate. In addition, it can inform compassion‐based trainers and course participants by elucidating the rationale behind the efficacy of such interventions (Carey et al. 2020).

### 1.1 Definitions of Self‐Compassion

Several complementary definitions and conceptual models exist regarding what self‐compassion is. A widely recognized conceptualisation is Neff's (2003a) model of self‐compassion. Neff states that self‐compassion comprises three key elements: (1) being kind and supportive to oneself, rather than being harsh and self‐judgemental, (2) recognising that difficulties constitute a normal part of human life, rather than feeling isolated from other people as a result of one's imperfections, and (3) keeping the personal suffering in mindful awareness, rather than becoming fully over‐identified and absorbed by one's problems (Neff 2003b). Other researchers have related self‐compassion to the general concept of compassion: a cognitive, affective, and behavioural process in response to suffering (Gilbert 2014, 2020). For instance, Strauss et al. (2016) identified five facets of compassion for oneself and others: (a) recognising suffering, (b) understanding the universality of human suffering, (c) feeling for the person suffering and emotionally connecting with the distress, (d) tolerating uncomfortable feelings in response to the suffering, remaining accepting of and open to the experience of suffering, and (e) acting or a motivation to act to alleviate suffering.

Conceptual models of self‐compassion have identified two potential pathways between self‐compassion and mental health: (1) emotion‐regulation and coping, and (2) self‐concept. In the section below, these constructs and their potential role in the association between self‐compassion and psychological outcomes are described.

### 1.2 Self‐Compassion, Emotion Regulation and Coping

First, it has been posited that the psychological benefits of self‐compassion can be explained by the strategies that people use to regulate their emotions and cope with stress (Ewert et al. 2021). *Emotion regulation* has been defined as the process of how we influence the type of emotion, the time that we have emotions, and how we experience and express emotions (Gross 2015). *Coping* refers to the cognitive and behavioural efforts to reduce the impact of stressful events and manage negative emotions, including stress, by altering the situation and/or tolerating or reducing negative emotions (Lazarus and Folkman 1984). Thus, while emotion regulation relates to managing emotions in various daily circumstances (not just unpleasant situations), coping specifically relates to managing stressful events and corresponding negative emotions (Trudel‐Fitzgerald et al. 2023).

Evidently, there is a degree of overlap between these two constructs. A comprehensive meta‐analysis examining the structure of emotion regulation and coping concluded that the 10 selected and most common emotion and coping strategies are highly related to each other (Naragon‐Gainey et al. 2017). Similarly, a conceptual scoping review highlighted similarities in the structure and content of self‐reported measures for coping and emotion regulation (Trudel‐Fitzgerald et al. 2023). Therefore, in this review, we combined emotion regulation and coping when summarising their role in the relationship between self‐compassion and psychological outcomes.

Emotion regulation and coping strategies can be divided into *adaptive* (e.g., problem‐solving, acceptance, positive reappraisal) and *maladaptive* strategies (e.g., avoidance, suppression, and rumination) (for an extensive review, see Naragon‐Gainey et al. 2017). While this generally holds, it should be mentioned that, according to the stress‐coping model (Lazarus and Folkman 1984), the efficacy and success of specific emotion regulation skills and coping strategies are likely to vary depending on the specific context, the individual involved, and their unique goals. Therefore, the ability to switch between different strategies in response to the precise demands of a situation is regarded as essential for effective coping (Trudel‐Fitzgerald et al. 2023).

In the past years, mindfulness also has been considered as an adaptive emotion regulation strategy (Naragon‐Gainey et al. 2017). *Mindfulness* refers to a purposeful, present‐moment awareness, with a nonjudgmental, open, and accepting attitude (e.g., without suppressing or becoming overwhelmed) (Kabat‐Zinn 2003). Self‐compassion encompasses elements of mindfulness, as the first step in being compassionate is to be aware and acknowledge suffering, with an open, nonjudging attitude and non‐reactivity (Gilbert 2020; Neff 2003a; Strauss et al. 2016). Obviously, mindfulness and self‐compassion share elements like non‐judgemental awareness, yet they can be seen as distinct and complementary (Brach 2004). Mindfulness relates to present‐moment awareness of all experiences (i.e., positive, negative, or neutral, not just of suffering as self‐compassion), whereas self‐compassion is broader than mindfulness and also relates to the sense of shared human experience and intention to relieve suffering. Acknowledging these conceptual notions, this review will regard mindfulness as a form of adaptive emotion regulation (Naragon‐Gainey et al. 2017).

Emotion regulation and coping mechanisms may constitute a central pathway through which self‐compassion influences mental health outcomes. Within Gilbert's (2020) well‐known theory of self‐compassion, psychological distress can be understood as the result of the hyperactivation of the threat and drive systems, whereas self‐compassion facilitates emotional regulation via activation of the affiliative or soothing system. Converging empirical findings provide physiological evidence suggesting that self‐compassion may be associated with increased parasympathetic activity and attenuated sympathetic nervous system activation (Di Bello et al. 2020).

Empirical evidence for the association of self‐compassion with emotion regulation and coping comes from a narrative overview by Finlay‐Jones (2017). Results showed that self‐compassion is linked to greater emotional awareness, acceptance, and clarity, as well as to less use of maladaptive strategies such as avoidance, rumination, and worry. A more recent meta‐analytical review on the relationship between self‐compassion and coping, across 136 studies involving 38,913 participants, also demonstrated positive correlations between self‐compassion and adaptive coping, and negative correlations with maladaptive, emotion‐focused coping (Ewert et al. 2021). Moreover, a systematic review by Inwood and Ferrari (2018) highlighted evidence suggesting that emotion regulation significantly mediates the association between self‐compassion and mental health. While these reviews have greatly contributed to our understanding of the mediators of self‐compassion, their findings are based on a limited number of studies. Inwood and Ferrari (2018) included only five cross‐sectional studies, with narrow types of outcomes and mediators (e.g., only negative outcomes and global emotion regulation). Moreover, the review covered studies published from 2011 until 2017 only. Since then, there has been a sharp increase in the number of studies in this field, warranting an updated review.

### 1.3 Self‐Compassion and Self‐Concept

Beyond influencing the use of particular emotion‐regulation and coping strategies, self‐compassion may affect psychological outcomes through our self‐concept. The *self‐concept* can be defined as a multifaceted structure, containing both stable and dynamic properties, regarding how individuals think about, evaluate, and relate to themselves, across time and social contexts (Cervone 2004; Mattingly et al. 2020). The self‐concept constitutes a content‐rich organizational structure encompassing self‐relevant knowledge that serves to guide, direct, and influence agentic functions of the self, including goal setting, motivation, and the regulation of emotion and behaviour (Mattingly et al. 2020; Talaifar and Swann 2018). A well‐integrated and balanced self‐concept is closely associated with better mental health and serves as a protective factor against psychological distress (Alessandri et al. 2021; Lee‐Flynn et al. 2011).

Self‐compassion, by encompassing less self‐criticism and more self‐kindness, may help individuals to obtain and maintain a clear, coherent, and stable understanding of their self‐concept, in times of failure or suffering (Neff 2003a, 2023b). Rather than excluding negative self‐relevant information or responding with harsh self‐criticism in such circumstances, self‐compassion may allow individuals to acknowledge imperfections and failures as part of a broader, balanced understanding of the self (Germer and Neff 2013; Miyagawa 2024; Neff 2003a, 2023b).

Empirical findings indicate that higher levels of self‐compassion are significantly associated with greater authenticity (Chew and Ang 2023) and lower levels of contingent self‐worth (Neff et al. 2018) and self‐stigma (Hilbert et al. 2015). Taken together, self‐concept may serve as a key pathway through which self‐compassion confers its psychological benefits, by fostering a more stable, authentic, and integrated sense of self in the face of adversity.

In summary, given the evidence for the psychological benefits of self‐compassion and compassion‐based interventions, we now need evidence to help us better understand why or how self‐compassion is beneficial for our psychological functioning. The aim of this systematic review is to synthesise and critically evaluate empirical studies that have investigated potential mediators of the relationship between self‐compassion and psychological outcomes. We hereby distinguish the role of potential beneficial and less beneficial mediating factors and positive outcomes (e.g. well‐being) versus negative outcomes (e.g. stress, depression). To obtain a comprehensive understanding, we adopted an inclusive approach by summarising the findings of all studies that examined mediators of the association of self‐compassion with psychological functioning, extending beyond the theorized constructs related to emotion regulation, coping, and self‐concept. Consequently, the mediational role of other factors including personal coping resources (e.g., optimism; Zhao et al. 2022) and social coping resources (e.g., social support; Allen et al. 2024) will also be considered.

## 2 Method

### 2.1 Search Procedure

This systematic review was conducted according to the PRISMA guidelines (Page et al. 2021) and registered at PROSPERO (CRD42022343868). The initial literature search was conducted using the electronic literature databases Web of Science, PsycINFO, PubMed and Embase in August 2022. Our search was informed by a previous review including the bibliometric analysis of the literature on self‐compassion in the past 2 decades (Swami et al. 2021). Three search sets were used which were linked with the Boolean operator “AND”. The first set related to self‐compassion and included the following search terms: “self compassion” OR self‐compassion. The second search set related to psychological outcomes and included the following search terms: “psychological health” OR “psychological symptoms” OR “psychiatric disorder” OR psychopathology OR “mental health” OR depress* OR anxiety OR stress OR distress OR burnout OR burn‐out OR wellbeing OR “well‐being” OR affect OR “post‐traumatic stress” OR “posttraumatic stress” OR “post‐traumatic growth” OR “posttraumatic growth” OR happiness. The third search set related to mechanism and included the following search terms: mediat* OR mechanism OR process. The search terms were entered for comprehensive searching in 'All fields' of articles. Similar to previous systematic reviews on self‐compassion (Cha et al. 2022), grey literature was not included, as it remains difficult to verify the completeness of including grey literature (e.g., unpublished thesis) and the quality of the research has not been evaluated by a peer‐review process. As the initial literature search was somewhat outdated at the time of manuscript revision, a second search was conducted, using the same electronic databases and the same search terms, to include newly published studies from August 2022 to January 2025. This literature search was expanded by checking the reference lists of three previous self‐compassion reviews for potential eligible studies for our review (Cha et al. 2022; Ewert et al. 2021; Inwood and Ferrari 2018). To reduce the potential risk of bias, we also conducted searches in August 2022 on the EU Clinical Trials Register (EU‐CTR), Australian New Zealand Clinical Trials Registry (ANZCTR), and Chinese Clinical Trial Registry (ChiCTR) to identify ongoing studies. Only four relevant registered trials could be identified, yet they were excluded as they focused on physiological and not psychological mediators. Similar to the literature search, we also conducted a second search in the registered trials database. Two additional registered trials investigating physiological factors as potential mediators were identified but excluded based on previously established criteria. Similarly, three trails that examined psychological factors as mediators between interventions (not self‐compassion) and psychological outcomes were also excluded.

### 2.2 Inclusion Criteria

Studies were included based on the following inclusion criteria: (1) published in peer‐reviewed journals, (2) written in English, (3) employed a quantitative methodology, (4) recruited adult samples, (5) utilised self‐report item(s) or questionnaires for assessing self‐compassion, psychological outcome(s), and potential mediator(s) (e.g., compassion‐based intervention studies using the intervention condition as an indicator of self‐compassion could therefore not be included, if not including a self‐report assessment of self‐compassion), (6) examined mediators of the relationship between self‐compassion and psychological outcomes.

**FIGURE 1:**
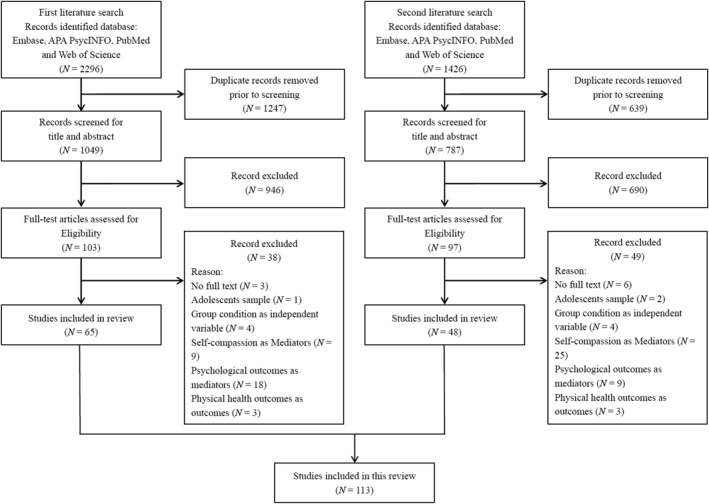
Flowchart of the literature search and article selection.

All references obtained from the initial search were exported to an Excel document after the removal of duplicates. The main author (JW) and the second reviewer (MK) screened the title and abstract of each of the 1049 papers for possible eligibility independently (see Figure 1 for a flowchart of study inclusion). Initially, two reviewers disagreed on the selection of 37 studies. After engaging in a thorough discussion focussing on the titles and abstracts, agreement was reached to retain five studies. These studies were found to have insufficient detail in their abstracts, necessitating a full‐text screening for a more comprehensive evaluation. Conversely, a consensus was reached to exclude 32 studies following the discussion. The full text of the 103 potentially eligible studies was read and screened against the eligibility criteria independently by both the main author and second reviewer. During the full‐text screening, reviewers rendered differing judgements on seven studies. After a thorough discussion, two of these studies were included, while three were excluded. The reviewers were unable to reach a consensus on the remaining two studies; hence, a third reviewer (MS) was consulted to make the final decision. Given that these two studies considered psychological outcomes (affect) as mediators, they were ultimately excluded. As with the initial literature screening, all references identified in the second search were exported to an Excel file after removing duplicates and studies already included in the initial search. Two reviewers (first author, JW; fourth reviewer, LC) independently screened the titles and abstracts of 787 records for potential eligibility (see Figure 1 for the study inclusion flowchart). Initial disagreements emerged on 25 studies. Following a detailed discussion centred on the relevance and clarity of titles and abstracts, consensus was reached to retain 12 studies for full‐text screening due to insufficient detail in their abstracts. The remaining 13 studies were excluded based on mutual agreement. Subsequently, the full texts of 97 potentially eligible studies were independently assessed against the eligibility criteria by the first author and the fourth reviewer. Discrepancies arose in the evaluation of five studies; after further deliberation, one study was included and four were excluded based on consensus.

### 2.3 Quality Assessment

The 11‐item Mediation Quality Checklist tool, initially formulated by Lubans et al. (2008) and adapted by Cerin et al. (2009) and Rhodes and Pfaeffli (2010), was employed to assess the mediation study quality of empirical studies, regarding scale reliability, mediation analysis methods, and assessment of change in mediators preceding change in the outcome (for intervention studies). All 11 items were used in the current review; the wording of some items was slightly adapted to align with our research questions (e.g., the dependent variable was specified as “psychological outcomes” in our evaluation). Each item can be scored in a yes (1) or no (0) format and a total score was generated by summing up all items. Following Cerin et al. (2009), studies scoring 0–3 were considered low‐quality, four to six medium‐quality, and seven to nine high‐quality. We classified studies scoring 10 and 11 as excellent, in the absence of previous labels. The primary author (JW) and the second reviewer (MK) independently assessed the mediation quality of all 65 studies identified in the first literature search, achieving an inter‐rater agreement of 90.77%. Discrepancies in 9.23% of cases (6 out of 65) were resolved either through mutual consensus (*n* = 4) or by consulting a third reviewer (MS) (*n* = 2), resulting in 100% agreement. Similarly, for the 48 studies identified in the second literature search, the primary author (JW) and the fourth reviewer (LC) conducted independent assessments, with an agreement rate of 85.42%. All disagreements (14.58%, *n* = 7) were resolved through mutual consensus, achieving full agreements.

### 2.4 Data Extraction

For the narrative summaries of the mechanisms, the following data were extracted from the included studies: authors, title, year, journal, sample characteristics, study design, mediator(s), outcome(s), measurement instruments for self‐compassion, mediator(s) and psychological outcome(s), type of mediation analysis, indirect effect, 95% confident interval (or *p*‐value if 95% CIs were not applicable), correlations between self‐compassion, mediator(s) and psychological outcomes, and indirect effect size (in instances where the original study did not provide an indirect effect size, it is computed as the ratio of the indirect effect to the total effect).

## 3 Results

### 3.1 Overview of the Studies

The first literature search yielded 2296 articles from Embase, APA PsycINFO, PubMed and Web of Science, of which 1247 were duplicates (see Figure 1, flowchart of the Literature Search and Article Selection). Of the remaining 1049 studies, 946 were excluded based on title and abstract screening. Subsequently, the full‐text review of 103 articles was conducted, and 65 articles from the first literature search met all inclusion criteria and were included in this review. In addition, the second literature search identified 1426 articles from the same databases. Of these, 639 were duplicates or overlapped with the results of the first search. After removing duplicates, 787 unique articles remained. Following the title and abstract screening, 690 articles were excluded. Full‐text assessments were subsequently conducted for the remaining 97 articles. Of these, 48 studies identified through the second search met all inclusion criteria and were included in the final review. Notably, Ewert et al. (2024) included two distinct studies in one paper, while the two papers by Maddock (2024a), (2025) and those by Kaçar‐Başaran (2025) and Kaçar‐Başaran and Gökdağ (2025), which drew on the same datasets respectively, were each counted as one study.

Accordingly, a total of 113 studies were included in this systematic review (see Appendix A for an overview of the 113 studies). Most studies (*n* = 91/113) employed a cross‐sectional observational design. Complementary to this, a smaller subset utilised longitudinal designs (*n* = 15/113; Allen et al. 2024; Asselmann et al. 2024; Cabaços et al. 2023; Diedrich et al. 2017; Ewert et al. 2024; Hsieh et al. 2021; Lefebvre et al. 2021; Maddock et al. 2020; Mosewich et al. 2019; Peng and Ishak 2024; Shi et al. 2025; Townshend and Caltabiano 2019; Ueno and Amemiya 2024; Zhang et al. 2025), diary design (1/113; Ewert et al. 2022), experimental design (*n* = 1/113; Ewert et al. 2018) or intervention design (*n* = 5/113; Kreemers et al. 2020; Maddock 2024b; Wadsworth et al. 2018; Xu et al. 2024; Yela et al. 2022).

Regarding the studied sample, 86 of the studies were conducted within the general population, while 11 studies in people with a psychological condition (Bakker et al. 2019; Cai et al. 2023; Carona et al. 2022; Chase et al. 2019; Diedrich et al. 2017; Eichholz et al. 2020; Krieger et al. 2013; Makadi and Koszycki 2020; Norman et al. 2020; Wadsworth et al. 2018; Xu et al. 2024), 11 studies in people with a physical condition (Carvalho et al. 2018; Cutajar and Bates 2025; Eghbali et al. 2022; Maddock et al. 2020; Morgenroth et al. 2022; Özönder Ünal et al. 2023; Ozonder Unal and Ordu 2023; Salehi et al. 2023; Skinner and Kuijer 2024; Zhu et al. 2020, 2022), and 5 studies in people exposed to trauma events (Allen et al. 2024; Blankenship and Hogge 2024; Hamrick and Owens 2019; Lenferink et al. 2017; Sperandio et al. 2022).

Regarding the assessment of self‐compassion, there was considerable homogeneity, with 111 of the 113 studies utilising a form of the Self‐Compassion Scale (SCS; Neff 2003b; Raes et al. 2011). The Compassionate Engagement and Action Scale (CEAS; Gilbert et al. 2017) was employed by two studies (Hsieh et al. 2021; Murfield et al. 2020).

### 3.2 Quality Assessment

The quality of mediation approaches was assessed for all 113 included studies (see Appendix B), with scores ranging from 1 to 10 out of 11. One study was rated as excellent (Ewert et al. 2024), 19 as high quality, 91 as medium quality, and 2 as low quality (Özönder Ünal et al. 2023; Ozonder Unal and Ordu 2023). Aside from general quality issues, 31 out of 113 studies lacked a well‐organised theoretical framework for their mediation model.

### 3.3 Overview of Mediators in the Relationship Between Self‐Compassion and Outcomes

More studies focused on negative psychological outcomes (93 assessments, e.g., negative affect, depression, anxiety, stress, distress, PTSD, grief, anger, burnout) than positive outcomes (44 assessments, e.g., life satisfaction, well‐being, positive affect, flourishing, post‐traumatic growth (PTG)). As can be seen in Tables 1 and 2, we found a wide range of mediators that were studied in the relationship between self‐compassion and these psychological outcomes. Emotion regulation and coping was the most studied mediator. Below, we will first describe the studied mediators in relation to negative psychological outcomes, followed by a description of the mediators related to positive psychological outcomes. Mediators were categorised into three groups: (1) emotion regulation/coping, (2) self‐concept, and (3) other.

**TABLE 1:** Mediators of the relationship between self‐compassion and negative outcomes.

Mediator (# studies)	Outcomes (refs)	Significant in single mediation model	Significant in multiple mediation model
Coping and emotion regulation			
*Generic strategies*			
Global emotion regulation (5)	*Depression* (Cutajar and Bates 2025; Diedrich et al. 2017; Ozonder Unal and Ordu 2023; Peng and Ishak 2024) *Anxiety* (Cutajar and Bates 2025) *Negative affect* (Ericson et al. 2024)	5/5	1/1
Emotion regulation difficulties (9)	*OCD severity* (Chase et al. 2019; Eichholz et al. 2020) *Distress* (Carona et al. 2022; Murfield et al. 2020; Xu et al. 2024) *Stress* (Finlay‐Jones et al. 2015) *Depression* (Cai et al. 2023) *Anxiety* (Akdeniz and Birekul 2024; Cai et al. 2023; Zhang et al. 2025)	10/10	—
Coping (7)	*Negative affect* (* Asselmann et al. 2024; Ewert et al. 2022; Mosewich et al. 2019) *Depression* (Beato et al. 2021; Wang et al. 2024); *Anxiety* (Beato et al. 2021) *Stress* (* Asselmann et al. 2024; Beato et al. 2021) *PTSD* (Hamrick and Owens 2019; Zerach 2025)	3/4	4/7
Resilience (7)	*Depression* (Ozonder Unal and Ordu 2023; Pérez‐Aranda et al. 2021; Zhao et al. 2022) *Anxiety* (Pérez‐Aranda et al. 2021) *Distress* (Hatun and Kurtça, 2022; Hou et al. 2025; Kaya et al. 2024; Ueno and Amemiya 2024); *Stress* (Zhao et al. 2022)	3/4	5/5
*Specific maladaptive strategies*			
Rumination/Worry/Repetitive negative thinking (22)	*Depression* (Arimitsu and Hofmann 2015; Bakker et al. 2019; S. L. Brown et al. 2020; Hodgetts et al. 2021; Johnson and O’Brien 2013; Krieger et al. 2013; Lenferink et al. 2017; Maddock 2024a; Maddock et al. 2020; Raes 2010; Wadsworth et al. 2018) *Anxiety* (Arimitsu and Hofmann 2015; S. L. Brown et al. 2020; Casali et al. 2022; Jansen et al. 2021; Maddock 2024a; Maddock et al. 2020; Raes 2010; Wadsworth et al. 2018) *Fear* (Jansen 2021; Jansen et al. 2021; Zhu et al. 2022) *Stress* (Karataş and Tüccar 2025; Maddock 2024b, 2025; Rakhimov et al. 2023) *PTSD* (Lenferink et al. 2017) *Grief* (Lenferink et al. 2017) *Anger* (Fresnics and Borders 2017) *Distress* (Norman et al. 2020) *Burnout* (* Cabaços et al. 2023)	20/21	6/6
Experiential avoidance (4)	*Depression* (Adie et al. 2021; Bakker et al. 2019; Krieger et al. 2013; Yela et al. 2022) *Anxiety* (* Yela et al. 2022)	4/4	1/2
Expressive suppression (3)	*Anxiety* (Bates et al. 2021; McBride et al. 2022) *Phobia* (Bates et al. 2021)	—	3/3
Denial (1)	*Shame* (* Ewert et al. 2018)	1/1	—
Self‐blame (1)	*PTSD* (Hamrick and Owens 2019); *Depression* (Hamrick and Owens 2019)	—	2/2
Self‐criticism (1)	*Negative affect* (* Kreemers et al. 2020)	2/2	—
Counterfactual thinking (1)	*Depression* (Angus and Phillips 2021)	0/1	—
Preservation of negative emotion (1)	*Depression* (Vidal et al. 2024)	—	1/1
Overproduction (1)	*Depression* (Vidal et al. 2024)	—	1/1
*Specific adaptive strategies*			
Mindfulness (4)	*Depression* (* Hsieh et al. 2021; Townshend and Caltabiano 2019) *Anxiety* (Makadi and Koszycki 2020) *Phobia* (Makadi and Koszycki 2020) *Stress* (Gouveia et al. 2016)	2/3	2/2
Tolerance (2)	*Depression* (Stephenson et al. 2018) *Anxiety* (Stephenson et al. 2018) *Distress* ((Kaçar‐Başaran 2025) *OCD* (Kaçar‐Başaran and Gökdağ 2025)	2/2	1/2
Cognitive reappraisal (3)	*Depression* (Bakker et al. 2019) *Anxiety* (Bates et al. 2021; McBride et al. 2022) *Phobi*a (Bates et al. 2021)	0/1	1/3
Acceptance (2)	*Depression* (Bakker et al. 2019; Carvalho et al. 2018)	2/2	0/1
Decentering (1)	*Depression* (Morgenroth et al. 2022) *Anxiety* (Morgenroth et al. 2022)	2/2	—
Positive automatic thought (1)	*Anxiety* (Arimitsu and Hofmann 2015) *Depression* (Arimitsu and Hofmann 2015)	—	2/2
Savouring‐anticipating (1)	*Depression* (Phillips 2018)	—	1/1
*Self‐concept*			
Self‐esteem (2)	*Depression* (Johnson and O’Brien 2013; Shi et al. 2025)	2/2	1/1
Self‐worth (1)	*Depression* (Stephenson et al. 2018) *Anxiety* (Stephenson et al. 2018)	1/2	—
Self‐concept clarity (1)	*Depression* (Coutts et al. 2023)	2/2	—
Self‐stigma (1)	*Depression* (Eccles et al. 2023) *Anxiety* (Eccles et al. 2023) *Stress* (Eccles et al. 2023)	3/3	—
Integrative self‐knowledge (1)	*Depression* (Ghorbani et al. 2012) *Anxiety* (Ghorbani et al. 2012)	2/2	—
Internalized heterosexism (2)	*Depression* (Brown‐Beresford and mclaren, 2022)	2/2	—
*Other*			
Optimism (2)	*Depression* (Phillips 2018; Zhao et al. 2022); *Stress* (Zhao et al. 2022)	—	2/3
Cognition style (1)	*Depression* (Zhou et al. 2013) *Hopelessness* (Zhou et al. 2013)	2/2	—
Relatedness (psychological needs) (2)	*Burnout* (Gerber et al. 2021; Gerber and Anaki 2021)	0/2	—
Autonomy (psychological needs) (2)	*Burnout* (Gerber et al. 2021; Gerber and Anaki 2021)	0/1	1/1
Attitudes to ageing (1)	*Depression* (L. Brown et al. 2016)	1/1	—
Illness perception (1)	*Depression* (Zhu et al. 2020); *anxiety* (Zhu et al. 2020)	2/2	—
Balanced time perspective (2)	*Depression* (Phillips 2018; Pyszkowska et al. 2024)	1/1	1/1
Sense of coherence (1)	*Depression* (Ying 2009)	1/1	—
Rejection sensitivity (1)	*Loneliness* (Xie et al. 2023)	1/1	—
Social support (1)	*PTSS* (* Allen et al. 2024)	1/1	—

^*^ Longitudinal or intervention study.

**TABLE 2:** Mediators of the relationship between self‐compassion and positive outcomes.

Mediator (# studies)	Outcomes (refs)	Significant in single mediation model	Significant in multiple mediation model
*Coping and emotion regulation*			
Emotion regulation difficulties (4)	*Flourishing* (Carona et al. 2022) *Positive affect* (Ericson et al. 2024) *Life satisfaction* (Ericson et al. 2024) *Well‐being* (Blankenship and Hogge 2024; Ericson et al. 2024; Julian et al. 2025)	4/5	1/1
Coping (6)	*Positive affect* (^a^ Ewert et al. 2022; Li et al. 2021;^a^ Mosewich et al. 2019) *Well‐being* (^a^ Asselmann et al. 2024;^a^ Ewert et al. 2024)	1/3	2/4
Resilience (5)	*Well‐being* (Eghbali et al. 2022; Hatun and Kurtça, 2023b; Voon et al. 2022) *Fulfilment* (Bogerd et al. 2023) *Quality of life* (Skinner and Kuijer 2024)	2/2	2/3
*Maladaptive coping*			
Negative automatic thoughts (1)	*Life satisfaction* (Arimitsu and Hofmann 2015)	—	0/1
Rumination/Worry (2)	*Well‐being* (Maddock 2024a;^a^ Maddock et al. 2020) *Life satisfaction* (Shin 2019)	2/3	0/1
Experiential avoidance (1)	*Well‐being* (^a^ Yela et al. 2022) *PTG* (Özönder Ünal et al. 2023)	1/1	1/1
Expressive suppression (1)	*Mental health* (Rehman et al. 2024)	—	1/1
Cognitive fusion (1)	*PTG* (Özönder Ünal et al. 2023)	—	1/1
Intolerance (1)	*Well‐being* (Deniz 2021)	—	1/1
Self‐criticism (1)	*Positive affect* (^a^ Kreemers et al. 2020)	2/2	—
*Adaptive coping*			
Active coping (1)	*PTG* (Munroe et al. 2022)	—	1/1
Positive reframing (2)	*PTG* (Munroe et al. 2022; Wong and Yeung 2017)	—	2/2
Mindfulness (1)	*PTG* (Özönder Ünal et al. 2023)		1/1
Positive automatic thoughts (1)	*Life satisfaction* (Arimitsu and Hofmann 2015)	—	1/1
Acceptance (1)	*PTG* (Wong and Yeung 2017) *Life satisfaction* (Zipagan and Galvez Tan 2023)	—	1/2
Cognitive reappraisal (1)	*Mental health* (Rehman et al. 2024)	—	1/1
Instrumental support (1)	*PTG* (Munroe et al. 2022)	—	1/1
Forgiveness (1)	*Well‐being* (Roxas et al. 2019)	1/1	—
Gratitude (1)	*Well‐being* (Nguyen and Le 2021)	—	1/1
Savouring‐anticipating (1)	*Life satisfaction* (Phillips 2018)	—	1/1
*Self‐concept*			
Self‐efficacy (1)	*Quality of life* (Salehi et al. 2023)	1/1	—
Self‐concept clarity (1)	*Life satisfaction* (Coutts et al. 2023)	1/1	—
Internalized Homonegativity (1)	*Life satisfaction* (Chong and Chan 2023)	1/1	—
*Other*			
Hope (3)	*Life satisfaction* (Tran et al. 2024; Yang et al. 2016) *PTG* (Sperandio et al. 2022) *Well‐being* (Tran et al. 2024) *Flourishing* (Liu et al. 2024)	3/3	—
Meaning of life (2)	*Happiness* (Wu et al. 2022) *Life satisfaction* (Zipagan and Galvez Tan 2023)	1/1	1/1
Narcissism (1)	*Well‐being* (Quang et al. 2022)	—	1/1
Optimism (1)	*Life satisfaction* (Phillips 2018)	—	0/2
Balanced time perspective (2)	*Life satisfaction* (Phillips 2018) *Well‐being* (Pyszkowska and Rönnlund 2021)	1/1	1/1
Attitudes to ageing (1)	*Well‐being* (L. Brown et al. 2016)	1/1	—
Perceived control (1)	*Well‐being* (Li and Wang 2024)	1/1	—
Prosocial behaviour (1)	*Well‐being* (Zeng et al. 2023)	1/1	—
Social safeness (1)	*Flourishing* (^a^ Lefebvre et al. 2021)	1/1	—
Help‐seeking behaviour (1)	*Flourishing* (Min et al. 2022)	1/1	—
Non‐attachment (1)	*Peace* (Xie 2023)	1/1	—

^a^ Longitudinal or intervention study.

### 3.4 Emotion Regulation and Coping in Relation to Negative Outcomes

#### 3.4.1 General Indicators of Emotion Regulation and Coping

In total, 21 studies investigated general indicators of emotion regulation and coping as mediators, with 14 studies consistently reporting a significant mediating effect of these indicators in the association of self‐compassion with negative outcomes (Akdeniz and Birekul 2024; Cai et al. 2023; Carona et al. 2022; Chase et al. 2019; Cutajar and Bates 2025; Diedrich et al. 2017; Eichholz et al. 2020; Ericson et al. 2024; Finlay‐Jones et al. 2015; Murfield et al. 2020; Ozonder Unal and Ordu 2023; Peng and Ishak 2024; Xu et al. 2024; Zhang et al. 2025). The seven remaining studies investigated generic coping styles as mediators (e.g. problem‐focused, emotion‐focused, avoidance‐focused coping; engagement and disengagement coping). Findings were mixed. Seven mediation models were significant, showing that one or more coping styles was a significant mediator in the relationship between self‐compassion and negative outcomes (Asselmann et al. 2024; Beato et al. 2021; Hamrick and Owens 2019; Mosewich et al. 2019; Wang et al. 2024; Zerach 2025). In contrast, 4 models reported non‐significant mediation effects, including a non‐significant mediating role of functional, disengagement and engagement coping styles (Asselmann et al. 2024; Beato et al. 2021; Ewert et al. 2022; Hamrick and Owens 2019).

Resilience, defined as a generic adaptive response to adversity (Richardson, 2002; Zautra et al., 2010), was also frequently examined as a mediator, with significant evidence for a mediating role of resilience in the relationship between self‐compassion and psychological distress (Bogerd et al. 2023; Eghbali et al. 2022; Hatun and Kurtça 2023; Skinner and Kuijer 2024; Voon et al. 2022).

#### 3.4.2 Specific Maladaptive Emotion Regulation and Coping Strategies

In total, 22 studies tested the mediating role of repetitive negative thinking, worry, and rumination. To clarify, rumination and worry, both recognized forms of repetitive negative thinking, have been identified as risk factors for depression and anxiety (Taylor and Snyder 2021). We found robust evidence that higher self‐compassion was associated with fewer psychological symptoms, via less use of repetitive negative thinking (Cabaços et al. 2023; Wadsworth et al. 2018), rumination and worry (Arimitsu and Hofmann 2015; Bakker et al. 2019; S. L. Brown et al. 2020; Casali et al. 2022; Fresnics and Borders 2017; Hodgetts et al. 2021; Jansen 2021; Jansen et al. 2021; Jansen et al. 2021; Johnson and O’Brien 2013; Karataş and Tüccar 2025; Krieger et al. 2013; Lenferink et al. 2017; Maddock 2024a, 2024b, 2025; Maddock et al. 2020; Norman et al. 2020; Raes 2010; Rakhimov et al. 2023; Zhu et al. 2022).

A second mediator, experiential avoidance, was examined in four studies (Adie et al. 2021; Bakker et al. 2019; Krieger et al. 2013; Yela et al. 2022), with significant effects observed in all but one study, where it was modelled alongside rumination (Bakker et al. 2019).

Additional strategies such as expressive suppression (Bates et al. 2021; McBride et al. 2022), denial (Ewert et al. 2018), self‐blame (Hamrick and Owens 2019), self‐criticism (Kreemers et al. 2020), preservation of negative emotion and overproduction (Vidal et al. 2024) were also identified as significant mediators, although the limited number of studies precludes firm conclusions.

#### 3.4.3 Specific Adaptive Emotion Regulation and Coping Strategies

Mindfulness has been the most extensively investigated adaptive mediator of self‐compassion in relation to negative outcomes. Four mediation models demonstrated that more self‐compassion relates to less psychological symptoms via higher levels of mindfulness (Gouveia et al. 2016; Hsieh et al. 2021; Makadi and Koszycki 2020; Townshend and Caltabiano 2019).

Acceptance and tolerance were also identified as significant mediators (Bakker et al. 2019; Carvalho et al. 2018; Kaçar‐Başaran 2025; Kaçar‐Başaran and Gökdağ 2025; Stephenson et al. 2018), though their effects diminished in multiple mediation models (Bakker et al. 2019; Stephenson et al. 2018). Additionally, other cognitive mechanisms such as positive automatic thoughts (Arimitsu and Hofmann 2015), savouring‐anticipating (Phillips 2018), and decentering (Morgenroth et al. 2022) have all been reported as significant mediators. Findings regarding cognitive reappraisal, however, were mixed: while three studies examined this pathway (Bakker et al. 2019; Bates et al. 2021; McBride et al. 2022), only McBride et al. (2022) reported a significant mediation effect.

Notably, most findings above are based on cross‐sectional studies. A few longitudinal, experimental, and intervention studies supported the mediating roles of repetitive negative thinking, worry and rumination (Cabaços et al. 2023; Maddock 2024b; Maddock et al. 2020; Wadsworth et al. 2018), as well as experiential avoidance (Yela et al. 2022), denial (Ewert et al. 2018), self‐criticism (Kreemers et al. 2020) and mindfulness (Hsieh et al. 2021; Townshend and Caltabiano 2019). Furthermore, given that most included studies investigate emotion regulation and coping as primary mediators of self‐compassion, the findings consistently highlight emotion regulation difficulties, rumination, worry, experiential avoidance, and mindfulness as key mechanisms between self‐compassion and psychological distress across diverse populations (See Table 3).

**TABLE 3:** Emotion regulation skills and coping strategies as self‐compassion mediators with negative outcomes across samples.

	General population	Participants with psychological condition	Participants with physical condition	Participants with trauma events
Global emotion regulation (difficulty)	✓ (6)		✓ (2)	—
Rumination	✓ (10)	✓ (2)	✓ (3)	✓ (1)
Worry	✓ (8)	—	✓ (2)	—
Experiential avoidance	✓ (2)	✓ (2)	—	—
Expressive suppression	✓ (1)	✓ (1)	—	—
Mindfulness	✓ (2)	✓ (1)	✓ (1)	—
Acceptance	—	✓ (1)	✓ (1)	—
Cognitive reappraisal	^a^ ✓(1)	^b^ ✓ (2)	—	—

^a^ no mediation model is significant.

^b^ not all mediation models are significant.

#### 3.4.4 Self‐Concept in Relation to Negative Outcomes

Five studies have investigated self‐concept as a mediator, suggesting that self‐compassion alleviates psychological distress by promoting more positive self‐evaluations. Specifically, higher levels of self‐compassion were related to lower levels of psychological distress, via enhanced self‐worth (Stephenson et al. 2018), higher self‐esteem (Johnson and O’Brien 2013; Shi et al. 2025), greater self‐concept clarity (Coutts et al. 2023), more integrative self‐knowledge (Ghorbani et al. 2012) and reduced self‐stigma (Eccles et al. 2023). In addition, two studies focused on internalized heterosexism as a mediating factor among LGBT populations (Brown‐Beresford and McLaren 2022; Ristvej et al. 2024). Longitudinal support for these pathways was limited, with only one study (Shi et al. 2025) providing evidence for a significant mediating role of self‐concept over time.

#### 3.4.5 Other Mechanisms in Relation to Negative Outcomes

Other potential mechanisms including optimism (Phillips 2018; Zhao et al. 2022), cognition style (Zhou et al. 2013), attitude to ageing (L. Brown et al. 2016 Brown et al., 2016), illness perception (Zhu et al. 2020), balanced time perspective (Phillips, 2018; Pyszkowska et al. 2024), sense of coherence (Ying 2009), rejection sensitivity (Xie et al. 2023) and social support (Allen et al. 2024) have also been found to function as significant self‐compassion mediators. However, psychological need (including autonomy and relatedness) was not (Gerber et al. 2021; Gerber and Anaki 2021).

### 3.5 Emotion Regulation and Coping in Relation to Positive Outcomes

#### 3.5.1 General Indicators of Emotion Regulation and Coping

Studies on emotion regulation difficulties as a self‐compassion mechanism for positive outcomes largely mirror findings for negative outcomes. Five out of six mediation models showed that more self‐compassion related to more well‐being by fewer emotion regulation difficulties (Blankenship and Hogge 2024; Carona et al. 2022; Ericson et al. 2024; Julian et al. 2025). In contrast, coping showed mixed results, with only three of seven models reporting a significant mediation effect (Ewert et al. 2022; Li et al. 2021; Mosewich et al. 2019).

In line with findings related to negative psychological outcomes, resilience has also been identified as a mediating mechanism in the relationship between self‐compassion and positive outcomes, with four of five mediation models reporting significant mediating effects (Bogerd et al. 2023; Eghbali et al. 2022; Hatun and Kurtça 2023; Skinner and Kuijer 2024; Voon et al. 2022).

#### 3.5.2 Specific Maladaptive Emotion Regulation and Coping Strategies

We found little empirical support for a significant role of negative automatic thoughts, worry, and rumination as mediators of self‐compassion, with only two of five mediation models reporting significant effects (Maddock 2024a; Shin 2019). Several studies demonstrated significant mediation effects for experiential avoidance (Özönder Ünal et al. 2023; Yela et al. 2022), expressive suppression (Rehman et al. 2024), cognitive fusion (Özönder Ünal et al. 2023), intolerance (Deniz 2021), and self‐criticism (Kreemers et al. 2020).

#### 3.5.3 Specific Adaptive Emotion Regulation and Coping Strategies

Significant adaptive emotion regulation and coping strategies that mediated the association of self‐compassion with positive outcomes included active coping (Munroe et al. 2022), positive reframing (Munroe et al. 2022; Wong and Yeung 2017), mindfulness (Özönder Ünal et al. 2023), positive automatic thoughts (Arimitsu and Hofmann 2015), acceptance (Wong and Yeung 2017; Zipagan and Galvez Tan 2023), cognitive reappraisal (Rehman et al. 2024), forgiveness (Roxas et al. 2019), gratitude (Nguyen and Le 2021), savouring‐anticipating (Phillips, 2018), and instrumental support (Munroe et al. 2022).

#### 3.5.4 Self‐Concept in Relation to Positive Outcomes

Three cross‐sectional studies have investigated self‐concept as a mediator in the relationship between self‐compassion and positive psychological outcomes. Consistent with findings related to negative outcomes, these studies suggest that self‐compassion may promote well‐being by fostering greater self‐efficacy (Salehi et al. 2023), enhanced self‐worth (Coutts et al. 2023) and clearer self‐knowledge (Chong and Chan 2023). The predominance of cross‐sectional designs limits the ability to draw causal inferences.

#### 3.5.5 Other Mechanisms in Relation to Positive Outcomes

Several other constructs have been found to mediate the relationship between self‐compassion and positive outcomes, including hope (Sperandio et al. 2022; Tran et al. 2024; Yang et al. 2016), meaning in life (Wu et al. 2022; Zipagan and Galvez Tan 2023), narcissism (Quang et al. 2022), balanced time perspective (Phillips 2018), attitude to ageing (L. Brown et al. 2016), perceived control (Li and Wang 2024), prosocial behaviour (Zeng et al. 2023), social safeness (Lefebvre et al. 2021), help‐seeking behaviour (Min et al. 2022), and non‐attachment (Xie 2023). In contrast, optimism did not emerge as a significant mediator (Phillips 2018).

## 4 Discussion

This paper presents a comprehensive review of empirical evidence on the mechanisms underlying the association between self‐compassion and psychological outcomes. Among the identified mediators, the use of (mal)adaptive emotion regulation and coping strategies emerged as the most extensively investigated and significant pathway. When looking at specific strategies, repetitive negative thinking, such as rumination and worry, was most frequently examined and demonstrated a robust mediating effect on psychological symptoms. Experiential avoidance as well as mindfulness were also identified as significant mediators. By comparison, findings for other potential mediators should be interpreted with caution due to the limited number of available studies. Relatively few studies focused on positive outcomes and the factors that mediate the association of self‐compassion with positive mental health and well‐being. This limits the ability to draw definitive conclusions in this area. Furthermore, it is important to highlight that evidence supporting these mediators has been observed across diverse study populations, including individuals from the general population, individuals with psychological and physical health conditions, and those exposed to traumatic events. Although not the primary focus of the present review, the findings indicate that commonly examined mediators, including global emotion regulation, repetitive negative thinking, experiential avoidance, and mindfulness, consistently played the mediating role between self‐compassion and psychological symptoms. Notably, these mediating effects appear to be robust across varying contexts and populations, suggesting that these factors explaining the psychological benefits of self‐compassion are rather robust and independent of the specific population and circumstances.

An important result from our review is that most studies focused on the association of self‐compassion with psychological symptoms (e.g., anxiety, depression) as outcomes and underlying mediators, rather than mental health and well‐being. This result is consistent with bibliometric analyses of the self‐compassion literature (Swami et al. 2021). Given that compassion is inherently oriented towards alleviating suffering, it is unsurprising that much of the existing research focused on the association of self‐compassion with psychological symptoms. However, the dual model of mental health posits that mental illness and mental well‐being represent two distinct but related dimensions, suggesting that true mental health encompasses not only the absence of mental illness but also the presence of positive mental well‐being (Iasiello et al. 2020). Beyond its role as an antidote to distress, self‐compassion is recognized as a positive resource fostering adaptive outcomes such as resilience, life satisfaction, happiness, and overall well‐being (Ewert et al. 2021; Zessin et al. 2015). Based on the limited number of studies testing the mediators of the association of self‐compassion with indicators of such adaptive outcomes, further research is warranted to investigate the facilitating role of self‐compassion in enhancing well‐being and to clarify the mechanisms through which these benefits are achieved.

A second key finding is that emotion regulation and coping were the most extensively studied mediators. Our findings demonstrate that higher levels of self‐compassion reliably related to fewer psychological symptoms and higher well‐being, through emotion regulation capacities. These findings align with Gilbert's (2014) theoretical framework, which proposes that self‐compassion enhances emotional regulation by reducing an overactive threat and drive system and activating the affiliative system. The evidence regarding coping as a mediating mechanism was somewhat mixed. One possible explanation for this is that not all coping strategies that were studied are equally likely to relate to self‐compassion (Ewert et al. 2021). Also, compared to studies on emotion regulation, the studies on coping employed a greater variety of measures, which may have contributed to variability in the findings.

Repetitive negative thinking (including worry and rumination) was identified as a stable mechanism through which self‐compassion may influence psychological symptoms. A second important and significant mediator was experiential avoidance. These findings are consistent and add to previous meta‐analyses, which identified rumination, worry, and experiential avoidance as coping strategies most closely associated with self‐compassion. A plausible explanation is that self‐compassion requires active engagement with one's suffering. When individuals avoid or resist this, attentional resources may become focused on distress and reactive responses, impairing the capacity to adopt a balanced and compassionate stance. This dynamic may, in turn, contribute to over‐identification with negative thoughts and emotions (Neff 2023a).

Mindfulness and related factors including acceptance and tolerance were identified as adaptive factors that significantly mediate the relationship between self‐compassion and psychological symptoms. This shows that people who are more self‐compassionate are more likely to be mindful, accepting, and tolerant, and therefore experience diminished psychological distress. Mindfulness may enable the recognition of negative thoughts and emotions as transient mental events, fostering the perspective needed to respond with compassion and consider how to best support oneself. This courageous awareness forms the foundation upon which self‐compassion is built (Neff 2023a). In addition, compassion involves staying present with painful emotions rather than avoiding them or becoming overwhelmed, and emotional tolerance plays a critical role in the process by which self‐compassion reduces psychological symptoms (Gilbert 2023).

In addition to emotion regulation and coping, a positive self‐concept and reduced self‐stigma were examined as mechanisms through which self‐compassion supports psychological outcomes. The reviewed evidence indicated that self‐compassion relates to fewer psychological symptoms and greater well‐being, by fostering a healthy self‐concept. This finding may explain the results of previous studies showing that inflated, unrealistic self‐views and low self‐esteem are related to poor mental health (Neff 2023a, 2023b; Sedikides 1993). Self‐compassion involves relating to oneself with kindness and acceptance, particularly during times of failure or perceived inadequacy. Rather than rejecting negative self‐related information or responding with harsh self‐criticism, individuals high in self‐compassion are more likely to acknowledge their imperfections as part of a broader and balanced understanding of the self (Germer and Neff 2013; Neff 2023a). Beyond healthy self‐concept, self‐stigma mediated the relationship between self‐compassion and mental health. According to Wong et al. (2019), self‐compassion inhibits public stigma through the cultivation of more balanced self‐perceptions, enabling individuals to accept both positive and negative aspects of the self while learning to observe and release self‐stigmatising thoughts and behaviours. Additionally, by enhancing social support and encouraging help‐seeking behaviour, self‐compassion also prevents psychological distress via less self‐stigma (Vigna and Strauss 2023). Future studies are needed to clarify the roles of self‐concept and self‐stigma as mechanisms of self‐compassion, especially given the current evidence is limited and largely based on cross‐sectional designs.

Other potential mediators received less empirical evidence, including social factors such as social relatedness, social safeness and social support. A scoping review by Lathren et al. (2021) found significant associations of self‐compassion with indicators of social functioning and social relationships. Compassion may provide a sense of meaning, fulfilment, and connectedness in social interactions. Our review highlights a gap in our knowledge concerning the social and interpersonal effects of self‐compassion, which warrants further research.

As we conducted two rounds of literature searches, we identified several emerging trends in research on the mechanisms of self‐compassion. Although the second search (2022–2025) spanned a shorter timeframe, it revealed a noticeable increase in studies, many of which replicated similar mediation models across diverse populations or focused on specific contexts such as the COVID‐19 pandemic. There is also increasing interest in exploring self‐compassion as a promotive factor for positive psychological outcomes. While most existing studies still rely on cross‐sectional designs, a growing number are beginning to adopt longitudinal and experimental methods. In addition, the scope of mediation research has expanded beyond emotion regulation to include a broader range of mechanisms, such as self‐concept and social support.

### 4.1 Limitations and Recommendations for Future Research

Several limitations should be acknowledged when interpreting our findings. First, the predominance of cross‐sectional designs limited our ability to infer causal or temporal relationships (Maxwell and Cole 2007). Future studies employing longitudinal or experimental designs are needed to clarify the directionality and underlying mechanisms of these associations. Second, most studies relied on the Self‐Compassion Scale (SCS) (Neff 2003b) or its short form version (Raes et al. 2011). The SCS has faced criticism due to overlap between its negative subscales and measures of psychopathology (Lathren et al. 2021; MacBeth and Gumley 2012). While alternative tools like the Sussex‐Oxford Compassion Scales (Gu et al. 2020) and the Compassionate Engagement and Action Scale (Gilbert et al. 2017) exist, they remain underused. A more general conceptual problem in the field is the conceptual overlap of constructs related to self‐compassion, mindfulness, and emotion regulation (Gilbert 2020; Gu et al. 2015; Neff 2003a, 2003b). This may confound the interpretation of mediation effects. To address this problem, future research should employ clear operational definitions and refined measurement tools to distinguish self‐compassion from related constructs. Third, most studies only tested mediation after demonstrating significant bivariate associations. This may introduce potential reporting bias. Fourth, as we only included studies with a self‐report measure of self‐compassion, we excluded potential valuable studies that examined mechanisms of mindfulness‐ or compassion‐based interventions in which self‐compassion and other psychological processes were modelled as parallel mediators. Future intervention research should consider serial mediation models or change scores to better identify causal pathways. Fifth, most studies did not report mediation effect sizes, and those that did often reported only the ratio of the indirect to total effect. This lack of consistent reporting hinders meaningful comparison of mediation effects across studies. Lastly, unpublished or non‐peer‐reviewed work was not included, though this is unlikely to have significantly altered our conclusions given the breadth of the included studies.

## 5 Conclusion

This review highlighted that the relationship between self‐compassion and psychological outcomes, as demonstrated by a vast amount of research, can be explained by a lower use of maladaptive emotion‐regulation strategies such as rumination, worry and experiential avoidance and a greater use of mindfulness. To validate these findings, especially the temporal direction, more rigorously designed research is needed to examine if it is truly self‐compassion leading to less avoidance, rumination, and distress, and more mindfulness and acceptance or the other way around.

## Author Contributions

JW conducted the search, screening, and study quality assessment, wrote the manuscript and contributed to all manuscript revisions. CD structured the review and contributed to all manuscript revisions. MK and LC collaborated in study screening and study quality assessment. EB conceptualized and structured of the review and contributed to all manuscript revisions. MS collaborated in study screening and study quality assessment, structured the review and contributed to all manuscript revisions. All authors collaborated in reviewing and editing the final manuscript and approved it for submission.

## Conflicts of Interest

The authors declare no competing interests.

## Supporting information

Supporting Information S1

## Data Availability

Data sharing is not applicable to this article as no new data were created or analysed in this study.
