# Supplementary material for: The Mechanisms Underlying the Relationship Between Self‐Compassion and Psychological Outcomes in Adult Populations: A Systematic Review
Source: Stress Health. 2025 Jul 28;41(4):e70090. doi: 10.1002/smi.70090 (PMC12302336; doi:10.1002/smi.70090)
Supplement: Supplementary file 1 — Supporting Information S1 [file SMI-41-e70090-s001.docx]

***Appendix A: Characteristics of included studies***

| **Authors** | **Self-compassion Measurement** | **Mediators [Measurement tool]/Correlation between self-compassion and mediators** | **Psychological outcome [Measurement tool]/Correlation between self-compassion and psychological outcomes** | **Correlation between mediator and psychological outcomes** | **Sample** | **Study design** | **Data analysis** | **Indirect effect [95% CI]/Indirect effect size** |  |
| --- | --- | --- | --- | --- | --- | --- | --- | --- | --- |
| Diedrich et al., 2017 | Self-Compassion Scale | Emotion regulation skills [Emotion Regulation Skills Questionnaire]  *r* = 0.40** | Depression [Beck Depression Inventory-II]  *r* = -0.35*** | *r* = -0.49*** | 69 clinically depressed individuals | Longitudinal | PROCESS/Simple mediation model | ***Depression [outcome]***  Global emotion regulation skills: indirect effect = -0.16 [-0.34, -0.06]/ *K*^2^ = 0.16 |  |
| Peng & Ishak, 2024 | Self-Compassion Scale-Short Form | Emotion regulation [Cognitive emotion regulation questionnaire short form]  Self-warmth – Adaptive emotion regulation: *r* = 0.312**  Self-warmth – Maladaptive emotion regulation: *r* = -0.089**  Self-coldness – Adaptive emotion regulation: *r* = -0.075**  Self-warmth – Maladaptive emotion regulation: *r* = 0.300** | Depression [Beck depression inventory]  Self-warmth: *r* = -0.217**  Self-coldness: *r* = 0.268** | Adaptive emotion regulation: *r* = -0.198**  Maladaptive emotion regulation: *r* = 0.258** | 21,353 undergraduates | longitudinal | SEM | ***Depression [outcome]***  Self-warm - Adaptive emotion regulation - depression: Indirect effect = -0.014 [-0.015, -0.012];  Self-warm - Maladaptive emotion regulation - depression: Indirect effect = -0.005 [-0.006, -0.002];  Self-coldness - Adaptive emotion regulation - depression: Indirect effect = 0.004 [0.003, 0.005];  Self-coldness - Maladaptive emotion regulation - depression: Indirect effect = 0.016 [0.014, 0.018]; |  |
| Ericson et al., 2024 | Self-Compassion Scale-Short Form | Emotion regulation [The Difficulties in Emotion Regulation Scale] *r* = -0.76** | Affect [The Positive and Negative Affect Schedule]  Positive affect: *r* = 0.44**  Negative affect: *r* = -0.64**  Life satisfaction [the Satisfaction With Life Scale] *r* = 0.46**  Eudemonic well-being [The Warwick-Edinburgh Mental Well-being Scale] *r* = 0.63** | Positive affect: *r* = -0.41**  Negative affect: *r* = 0.70**  Life satisfaction: *r* = -0.40**  Eudemonic well-being: *r* = -0.58** | 559 adults | Cross-sectional | Mediation analysis | ***Positive affect [outcome]***  Indirect effect = 1.39 [0.34, 2.46]/ *R*^2^ = 0.24  ***Negative affect [outcome]***  Indirect effect = -4.14 [-5.19, -3.08]/ *R*^2^ = 0.54  ***Life satisfaction [outcome]***  Indirect effect = 0.7 [-0.27, 1.65]  ***Eudemonic well-being [outcome]***  Indirect effect = 2.44 [1.14, 3.75]/ *R*^2^ = 0.44 |  |
| Ozonder Unal & Ordu, 2023 | Self-Compassion Scale | Emotion regulation [Cognitive Emotion Regulation Questionnaire] Adaptive emotion regulation: *r* = 0.35**; Maladaptive emotion regulation: *r* = -0.22**  Resilience [the Brief Resilience Scale] *r* = 0.21** | Depression [Beck Depression Scale]  *r* = -0.43** | Adaptive emotion regulation: *r* = -0.40**; Maladaptive emotion regulation: *r* = 0.40**；  Resilience: *r* = -0.27** | 151 stage 4 cancer patients | Cross-sectional | Mediation analysis | ***Depression [outcome]***  Adaptive emotion regulation: Indirect effect = -0.079 [-0.294, -0.042] *PM* = 0.18.  Maladaptive emotion regulation: Indirect effect = -0.109 [-0.212, -0.020] *PM* = 0.25.  Resilience: Indirect effect = -0.032 [-0.141, -0.003] *PM* = 0.07. |  |
| Cutajar & Bates, 2025 | Self-Compassion Scale-Short Form | Emotional Regulation [Emotion Regulation Questionnaire; Brief COPE scale; Difficulties in Emotion Regulation Scale; Cognitive Emotion Regulation Questionnaire] | Anxiety/Depression [The Hospital Anxiety and Depression Scale] | Not applicable | 265 Australian perinatal women | Cross-sectional | Mediation analysis | ***Depression [outcome]***  Emotion regulation: indirect effect = -0.29 [-0.42, -0.16]/*PM* = 0.52  ***Anxiety [outcome]***  Emotion regulation: indirect effect = -0.37 [-0.51, -0.25]/*PM* = 0.66 |  |
| Eichholz et al., 2020 | Self-Compassion Scale-Short Form | Emotion regulation difficulty [Difficulties in Emotion Regulation Scale]/  *r* = -0.61*** | Obsessive-compulsive symptom severity [Yale-Brown Obsessive Compulsive Scale]/  *r* = -0.40*** | *r* = 0.49*** | 90 OCD adult patients | Cross-sectional | PROCESS/Simple mediation model | ***OCD severity [outcome]***  Emotion regulation difficulties: indirect effect = -1.95 [-3.65, -0.74] (Unstandardized)/ *PM* = 0.60 |  |
| Chase et al., 2019 | Self-Compassion Scale | Emotion regulation difficulty [Difficulties in Emotion Regulation Scale]/  *r* = -0.74** | Obsessive-compulsive symptom severity [Dimensional Obsessive-Compulsive Scale]/  *r* = -0.44** | *r* = 0.50** | 62 OCD patients | Cross-sectional | PROCESS/simple mediation model | ***OCD severity [outcome]***  Emotion regulation difficulties: indirect = -0.27 [-0.52, -0.04] (Unstandardized)/*PM* = 0.77 |  |
| Murfield et al., 2020 | Compassionate Engagement and Action Scales | Emotion regulation difficulty [Difficulties in Emotion Regulation Scale]/ *r* = -0.59** | Distress [Depression Anxiety Stress Scales – 21]/  Depression: *r* = -0.46**  Anxiety: *r* = -0.34**  Stress: *r* = -0.38** | Depression: r = 0.69**  Anxiety: *r* = 0.59**  Stress: *r* = 0.70** | 141 family caregivers of older adults | Cross-sectional | AMOS/SEM | ***Distress [outcome]***  Emotion regulation difficulties: indirect effects is significant [parameters undisclosed]/Not applicable |  |
| Finlay-Jones et al., 2015 | Self-Compassion Scale-Short Form | Emotion regulation difficulty [Difficulties in Emotion Regulation Scale]/ *r* = -0.56*** | Stress [Depression Anxiety Stress Scales – 21, only stress subscale]/ *r* = -0.37*** | *r* = 0.48*** | 198 psychologists | Cross-sectional | LISREL/SEM | ***Stress [outcome]***  Emotion regulation difficulties: *z*’ = 3.83, *p* < 0.001./Not applicable |  |
| Carona et al., 2022 | Self-Compassion Scale-Short Form | Emotion regulation difficulty [Difficulties in Emotion Regulation Scale]/ *r* = -0.71* | Distress  Flourishing  [Edinburgh Postnatal Depression Scale; Anxiety Subscale of the Hospital Anxiety and Depression Scale; Mental Health Continuum Short Form]/  Anxiety: *r* = -0.53*  Depression: *r* = -0.57*  Flourishing: *r* = 0.58* | Anxiety: *r* = 0.55*  Depression: *r* = 0.60*  Flourishing: *r* = -0.56* | 1053 postpartum women presenting high risk for PPD | Cross-sectional | AMOS/SEM | ***Distress [outcome]***  Emotion regulation difficulties: indirect effects = -0.335 [-0.380, -0.290]/*PM* = 0.53 ***Flourishing [outcome]:*** Emotion regulation difficulties: indirect effects = 0.229 [0.179, 0.271]/*PM* = 0.39 |  |
| Cai et al., 2023 | Self-Compassion Scale | Emotion regulation [Difficulties in Emotion Regulation Scale] *r* = -0.66** | Anxiety [Generalized Anxiety Disorder Dimensional Scale] *r* = -0.23*  Depression [Patient Health Questionnaire-9] *r* = -0.30** | Anxiety: r = 0.49**  Depression: *r* = 0.54** | 153 autistic adults | Cross-sectional | Mediation analysis | ***Anxiety [Outcome]***  Indirect effect = -0.18 [-0.25, -0.12]/ PM = 0.72  ***Depression [Outcome]***  Indirect effect = -0.14 [-0.20, -0.09]/PM = 0.82 |  |
| Akdeniz & Birekul, 2024 | Self-Compassion Scale | Emotion regulation difficulty [The Difficulties in Emotion Regulation Scale] *r* = 0.621** | Anxiety [Social Anxiety Scale] *r* = -0.474** | *r* = 0.612** | 975 young adults | Cross-sectional | Mediation analysis | ***Anxiety [outcome]***  Indirect effect = -0.06 [-0.08, -0.04]/*PM* = 0.29 |  |
| Zhang et al., 2025 | Self-Compassion Scale-Short Form | Emotion regulation difficulty [The Difficulties in Emotion Regulation Scale] | Anxiety | Not applicable | 4312 students | Longitudinal | Random intercept cross-lagged panel model | ***Anxiety [outcome]***  Indirect effect = -0.004 [-0.006, -0.002];  -0.004 [-0.006, -0.001];  -0.003 [-0.005, -0.001]; |  |
| Xu et al., 2024 | Self-Compassion Scale | Alexithymia [Toronto alexithymia scale] *r* = -0.51** | Psychosomatic distress [Brief symptom inventory-18] *r* = -0.68** | *r* = 0.57** | 116 participants with psychosomatic symptom distress | Intervention | Serial mediation model | ***Distress [outcome]***  Indirect effect = -0.621 [-1.013, -0.320]/*PM* = 0.15 |  |
| Pérez-Aranda et al., 2021 | Self-Compassion Scale-Short Form | Resilience [Connor-Davidson Resilience Scale]/Not applicable | Depression Anxiety [Goldberg Anxiety and Depression Scale]/  Depression: *r* = -0.31*  Anxiety: *r* = -0.31* | Not applicable | 860 subjects | Cross-sectional | Mplus/Path analysis | ***Anxiety [outcome]***  Resilience: indirect effect = -0.03 [-0.15, 0.09]/*R*^2^ = 0.25 ***Depression [outcome]***  Resilience: indirect effect = -0.19 [-0.33, -0.07] /*R*^2^ = 0.25 |  |
| Zhao et al., 2022 | Self-Compassion Scale-Short Form | Resilience Optimism [10‑item Connor‑Davidson Resilience Scale; Life Orientation Test]/  Resilience: *r* = 0.40** Optimism: *r* = 0.45** | Depression [Patient Health Questionnaire depression scale]/  Depression: *r* = -0.37** | Resilience - Depression: *r* = -0.39** Optimism - Depression: *r* = -0.33** | 565 medical students and 560 nursing students | Cross-sectional | AMOS/Path analysis | ***Depression (Nursing students) [outcome]*** Resilience: indirect effect = -0.108 [-0.151, -0.075] /Not applicable; Optimism: indirect = -0.086[-0.142, -0.042] /Not applicable; ***Depression (Medical students) [outcome]*** Resilience: indirect effect = -0.042 [-0.077, -0.017] /Not applicable; Optimism: indirect effect = -0.090 [-0.139, -0.052] /Not applicable. |  |
| Hatun & Kurtça, 2023 | Self-Compassion Scale-Short Form | Resilience [Brief Resilience Scale]/  *r* = 0.67*** | Psychological distress  Well-being [Patience Health Questionnaire;Psychological Well‑being Scale]/  Psychological distress: *r* = -0.63***  Well-being: *r* = 0.54*** | Psychological distress: *r* = -0.53***  Well-being *r* = 0.47*** | 617 adults | Cross-sectional | PROCESS/Serial mediation model | ***[serial mediation model]***  Self-compassion - Resilience - Well-being: indirect effect = 0.070 [0.006, 0.134]/*PM* = 0.12; Self-compassion - Resilience - Distress - Well-being: indirect effect = 0.038 [0.019, 0.061]/*PM* = 0.07 |  |
| Ueno & Amemiya, 2024) | Self-Compassion Scale | Resilience [The Adolescent Resilience Scale] *r* = 0.6** | Psychological distress [10-item Kessler Psychological Distress Scale] *r* =-0.47** | *r* = -0.52** | 486 participants | Longitudinal design for 2 years | SEM | ***Distress [outcome]***  Indirect effect = - 0.07 [-0.13, -0.02]/ Not applicable |  |
| Kaya et al., 2024 | Self-Compassion Scale | Resilience [The Brief Resilience Scale] *r* = 0.60** | Distress [Death Distress Scale] *r* = -0.37** | r = -0.42** | 364 adults | Cross-sectional | SEM | ***Distress [outcome]***  Significant [parameters undisclosed] |  |
| Hou et al., 2025 | Self-Compassion Scale | Resilience [Connor and Davidson’s Resilience Scale] | Distress [Depression, Anxiety, Stress Scale -21] | Not applicable | 2263 students | Cross-sectional | Mediation analysis | ***Distress [outcome]***  Compassionate self-responding - resilience - psychological distress: indirect effect = -0.04 [-0.06, -0.02]/ Not applicable  Uncompassionate self-responding - resilience - psychological distress: indirect effect = 0.02 [0.01, 0.03]/ Not applicable |  |
| Mosewich et al., 2019 | Self-Compassion Scale | Coping [Coping Function Questionnaire]  Problem-focused coping: *r* = 0.094  Emotion-focused coping: *r* = -0.057  Avoidance-focused coping: *r* = -0.254** | Affect [Positive and Negative Affect Schedule]  Positive affect: *r* = 0.157  Negative affect: *r* = -0.391 | Problem-focused coping – Positive affect: *r* = -0.056  Emotion-focused coping – Positive affect: *r* = 0.135  Avoidance-focused coping – Positive affect: *r* = -0.229**  Problem-focused coping – Negative affect: *r* = 0.105  Emotion-focused coping – Negative affect: *r* = 0.114  Avoidance-focused coping – Negative affect: r = 0.464** | 122 women athletes | Longitudinal | Mplus/SEM | ***[serial mediation model]***  Self-compassion - control appraisals - avoidance-focused coping - negative affect: indirect effect = -0.019 [-0.073, - 0.001]  Self-compassion - treat appraisals - avoidance-focused coping - negative affect: indirect effect = -0.020 [-0.067, - 0.001]  Problem-focused coping/Emotion-focused coping: indirect effects are not significant [parameters undisclosed]. |  |
| Beato et al., 2021 | Self-Compassion Scale | Coping [Brief-COPE]  Emotion coping: *r* = 0.407***  Problem coping: *r* = 0.342***  Dysfunctional coping: *r* = -0.358*** | Depression Anxiety Stress [The Short Version of Depression, Anxiety and Stress Scale]  Depression: *r* = -0.553***  Anxiety: *r* = -0.469***  Stress: *r* = -0.517*** | Emotional coping – Depression: *r* = -0.164**  Problem coping – Depression: *r* = -0.178***  Dysfunctional coping - Depression: *r* = 0.477***  Emotional coping – Anxiety: *r* = -0.130**  Problem coping – Anxiety: *r* = -0.058  Dysfunctional coping - Anxiety: *r* = 0.385***  Emotional coping – Stress: *r* = -0.109*  Problem coping – Stress: *r* = 0.022  Dysfunctional coping - Stress: *r* = 0.439*** | 428 Portuguese adults | Cross-sectional | PROCESS/Paralleled mediation model | ***Depression [outcome]*** Dysfunctional coping: indirect effect = -0.018 [-0.028, -0.010]/*PM* = 0.15 Problem-oriented coping: indirect effect = -0.010 [-0.019, -0.002]/*PM* = 0.08 Emotional coping: indirect effect = 0.005 [-0.004, 0.015]/*PM* = 0.04 ***Anxiety [outcome]*** Dysfunctional coping/Problem-oriented coping/Emotional coping: indirect effects are not significant [parameters undisclosed]. ***Stress [outcome]*** Problem-oriented coping: indirect effect = 0.01 [0.004, 0.023]/*PM* = 0.08 Dysfunctional coping /Emotional coping: indirect effects are not significant [parameters undisclosed]. |  |
| Ewert et al., 2022 | Self-Compassion Scale | Coping  [Brief COPE inventory]  Engagement coping: r = 0.32  Disengagement coping: r = -0.24 | Affect  [Six mood adjectives representing the structure of afect  (Blanke & Brose, 2017)]  Positive affect: r = 0.47  Negative affect: r =-0.48 | Engagement coping – Positive affect: r = 0.14  Engagement coping – Negative affect: r = -0.13  Disengagement coping – Positive affect: r = -0.24  Disengagement coping – Negative affect: r = 0.32 | 213 participants | 1-week ambulatory assessment study | Multilevel Mediation Analyses | ***Positive affect [outcome]***  Engagement coping: indirect effect = 0.042 [0.01, 0.08]/ Not applicable  Disengagement coping: indirect effect = 0.052 [-0.02, 0.12]/ Not applicable  ***Negative affect [outcome]***  Engagement coping: indirect effect = −0.025 [−0.06, 0.01]/ Not applicable  Disengagement coping: indirect effect = −0.043 [−0.10, 0.02]/Not applicable |  |
| Asselmann et al., 2024 | Self-Compassion Scale | Coping [the Brief COPE inventory] | Life satisfaction [The Satisfaction with Life Scale]  Affect [Positive and Negative Affect Schedule]  Psychological symptoms [The Depression Anxiety Stress Scale] | Not applicable | 430 adults | Longitudinal design for 6 weeks | SEM | ***Positive affect [outcome]***  Functional coping: Indirect effect = 0.03 [0.00, 0.07]/Not applicable  Dysfunctional coping: Indirect effect = [0.01, 0.06]/Not applicable  ***Negative affect [outcome]***  Functional coping: Indirect effect = -0.02 [-0.03, 0.00]/ Not applicable  Dysfunctional coping: Indirect effect = -0.04 [-0.07, -0.01]/*PM* = 0.31  ***Stress [outcome]***  Functional coping: Indirect effect = -0.03 [-0.05, 0.00]/Not applicable  Dysfunctional coping: significant: Indirect effect = -0.06 [-0.10, -0.01]/*PM* = 0.46 |  |
| Wang et al., 2024 | Self-Compassion Scale | Coping style [Simple coping style questionnaire] *r* = 0.61 | Depression [The Depression-Anxiety-Stress Scale] *r* = -0.22** | *r* = -0.45** | 1038 students | Cross-sectional | Mediation analysis | Indirect effect = -0.267 [-0.333, -0.208]/*PM* = 0.84 |  |
| Zerach, 2025 | Self-Compassion Scale | Coping [Brief COPE] adaptive coping: *r* = 0.24** (combat veterans)  *r* = 0.15** (noncombat veterans)  Maladaptive coping: *r* = -0.43** (combat veterans)  *r* = -0.24** (noncombat veterans) | PTSD [International Trauma Questionnaire for PTSD] *r* = -0.17** (combat veterans)  *r* = -0.27** (noncombat veterans) | Adaptive coping: *r* = 0.06 (combat veterans)  *r* = -0.01 (noncombat veterans)  Maladaptive coping: *r* = 0.35** (combat veterans)  *r* = 0.28** (noncombat veterans) | 885 combat veterans; 728 noncombat veterans | Cross-sectional | Mediation analysis | ***PTSD [outcome]***  Adaptive coping: indirect effect = -0.02 [-0.18 0.13]/Not applicable (combat veterans)  Indirect effect = -0.09 [-0.24 0.01]/Not applicable (Noncombat veterans)  Maladaptive coping: indirect effect = -0.95 [-1.27, -0.65] /Not applicable (combat veterans)  Indirect effect = -0.95 [-1.36, -0.55]/Not applicable  (Noncombat veterans) |  |
| Hamrick & Owens, 2019 | Self-Compassion Scale-Short Form | Self-blame Disengagement coping [Rape Attribution Questionnaire; Coping Strategies Inventory‐Short Form]/  Behavioral self‐blame: *r* = -0.36* Characterological self-blame: *r* = -0.41* Disengagement coping: *r* = -0.45* | Depression PTSD [Depression Anxiety Stress Scales‐21; PTSD checklist for DSM‐5]/  PTSD: *r* = -0.31*  Depression: *r* = -0.39* | Behavioral self‐blame - PTSD: *r* = 0.54* Characterological self-blame - PTSD: *r* = 0.59* Disengagement coping - PTSD: *r* = 0.48*  Behavioral self‐blame - Depression: *r* = 0.33* Characterological self-blame - Depression: *r* = 0.50* Disengagement coping - Depression: r = 0.30* | 207 females following the sexual assault | Cross-sectional | PROCESS/Paralleled mediation model | ***PTSD [outcome]***  Behavioral self‐blame: indirect effect = -0.152 [0.323, -0.021]/*PM* = 0.18;  Characterological self-blame: indirect effect = -0.359 [-0.565, -0.204]/*PM* = 0.43;  Disengagement coping: indirect effect = -0.280 [-0.467, -0.143]/*PM* = 0.34 (Unstandardized)  ***Depression [outcome]*** Characterological self-blame: indirect effect = -0.254 [-0.386, -0.148]/*PM* = 0.59 Behavioral self‐blame / Disengagement coping indirect effects are not significant [parameters undisclosed] |  |
| S. L. Brown et al., 2020 | Self-Compassion Scale | Rumination Worry [Ruminative Response Scale; Penn State Worry Questionnaire]/Not applicable | Depression Anxiety [Hospital Anxiety and Depression Scale] /Not applicable | Not applicable | 184 breast cancer survivors | Cross-sectional | AMOS/SEM | Self-compassion subscales were used. |  |
| Bakker et al., 2019 | Self-Compassion Scale | Rumination Experiential Avoidance Cognitive Reappraisal Acceptance [Ruminative Responses Scale; Brief Experiential Avoidance Questionnaire; Emotion Regulation Questionnaire; Difficulties in Emotion Regulation Scale]/  Rumination: *r* = -0.44** Experiential Avoidance: *r* = -0.40** Cognitive Reappraisal: *r* = 0.58** Acceptance: *r* = 0.44** | Depression [Beck Depression Inventory-II]/  *r* = -0.57** | Rumination: *r* = 0.54** Experiential Avoidance: *r* = 0.44** Cognitive Reappraisal: *r* = -0.41** Acceptance: *r* = -0.42 | 100 participants with a history of recurrent depression | Cross-sectional | PROCESS/Simple mediation model | ***Depression [outcome]***  Rumination: indirect effect = -0.16 [-0.30, -0.07]/*PM* = 0.28 Experiential avoidance: indirect effect = -0.10 [-0.22, -0.03]/*PM* = 0.18 Cognitive reappraisal: indirect effect = -0.07 [-0.21, 0.03]/*PM* = 0.12 Acceptance: indirect effect = -0.09 [-0.20, -0.02]/*PM* = 0.16 |  |
| Raes, 2010 | Self-Compassion Scale | Rumination Worry [Ruminative response scale; Penn state worry questionnaire]/  Rumination [Reflection]: *r* = -0.19**  Rumination [Brooding]: *r* = -0.55***  Worry: *r* = -0.62*** | Depression Anxiety [Beck depression inventory-II; State-trait anxiety inventory]/  Depression: *r* = -0.55***  Anxiety: *r* = -0.75*** | Rumination [Reflection] - Depression: *r* = 0.26***  Rumination [Brooding] - Depression: *r* = 0.57***  Worry - Depression: *r* = 0.47***  Rumination [Reflection] - Anxiety: *r* = 0.26***  Rumination [Brooding] - Anxiety: *r* = 0.62***  Worry - Anxiety: *r* = 0.68 | 271 psychology undergraduates | Cross-sectional | No report/Paralleled mediation model | ***Depression [outcome]*** Reflection (subscale of rumination): indirect effect = 0.00 [-0.014, 0.001]/*PM* = 0; Brooding (subscale of rumination): indirect effect = -0.07 [-0.100, -0.039]/*PM* = 0.37; Worry: indirect effect = -0.02 [-0.051, 0.006]/*PM* = 0.11; ***Anxiety [outcome]*** Reflection (subscale of rumination): indirect effect = 0.00 [-0.014, 0.004]/*PM* = 0; Brooding (subscale of rumination): indirect effect = -0.06 [-0.091, -0.029]/*PM* = 0.16; Worry: indirect effect = -0.08 [-0.116, -0.047]/*PM* = 0.22 |  |
| Maddock et al., 2020 | Self-Compassion Scale | Rumination Worry [Rumination Reflection Questionnaire; Penn State Worry Questionnaire]/Not applicable | Depression Anxiety [Hospital Anxiety and Depression Scale] /Not applicable | Not applicable | 285 psoriasis patients | Longitudinal | LISREL/SEM | ***Anxiety [outcome]***  Worry/Rumination: indirect effects are significant [parameters undisclosed] /Not applicable  ***Depression [outcome]***  Worry/Rumination: indirect effects are not significant [parameters undisclosed] /Not applicable  ***Well-being [outcome]***  Worry/Rumination: indirect effects are not significant [parameters undisclosed] /Not applicable |  |
| Jansen, 2021 | Self-Compassion Scale | Rumination Worry [Rumination-reflection questionnaire; Penn-State worry questionnaire]  Self-compassion[positive] – Rumination: *r* = -0.27*  Self-compassion[positive] – Worry: *r* = -0.33*  Self-compassion[negative] – Rumination: *r* = 0.57**  Self-compassion[negative] – Worry: *r* = 0.69** | Fear of the Future/  Self-compassion[positive] – Worry: *r* = -0.025  Self-compassion[negative] – Rumination: *r* = 0.433** | Rumination: *r* = 0.188  Worry: *r* = 0.528** | 55 athletes | Cross-sectional | PROCESS/Simple mediation model | ***Fear of future [outcome]***  Worry: indirect effect = 0.794 [0.325, 1.359] (Unstandardized)/*PM* = 0.53  Rumination): indirect effect = -0.372 [-0.751, -0.047] (Unstandardized)/*PM* = 0.24 |  |
| Jansen, Siebertz, et al., 2021 | Self-Compassion Scale | Rumination Worry [Rumination-reflection questionnaire; Penn-State worry questionnaire]/  Rumination: *r* = -0.53***  Worry: *r* = -0.57*** | Fear of the Future/  *r* = -0.28*** | Rumination: r = 0.30***  Worry: *r* = 0.41*** | 2765 adults | Cross-sectional | PROCESS/Paralleled mediation model | ***Fear of future [outcome]***  Worry: indirect = -0.46, [-0.53, -0.39]/*PM* = 0.74 Rumination: indirect effect is not significant [parameters undisclosed] |  |
| Rakhimov et al., 2023 | Self-Compassion Scale | Worry [Anxiety and Preoccupation about Sleep Questionnaire]/  *r* = -0.28*** | Stress [Perceived Stress Scale]/  *r* = -0.53*** | *r* = 0.57*** | 468 adults | Cross-sectional | AMOS/SEM | ***[serial mediation model]***  Self-compassion – Worry – Perceived stress – Poor sleep quality: indirect effect = -0.10, *p* > 0.05/ Not applicable  Self-compassion – Worry – Perceived stress – Poor sleep hygiene - Poor sleep quality: indirect effect = -0.10, *p* <0.01/ Not applicable |  |
| Lenferink et al., 2017 | Self-Compassion Scale | Rumination [Utrecht Grief Rumination Scale]/ *r* = -0.29** | Grief Depression PTSD [Inventory of Complicated Grief; Inventory of Depressive Symptomatology; PTSD Checklist for DSM-5]/  Grief: *r* = -0.35***  Depression: *r* = -0.41***  PTSD: *r* = -0.46*** | Grief: r = 0.79***  Depression: *r* = 0.64***  PTSD: *r* = 0.70*** | 137 relatives of long-term missing persons | Cross-sectional | PROCESS/Simple mediation model | ***Prolonged grief [outcome]***  Rumination: indirect effect = -0.11 [-0.02, -0.03]/*K*^2^ = 0.50 ***Depression [outcome]***  indirect effect = -0.07 [-0.13, -0.03]/ *K*^2^ = 0.32 ***PTSD [outcome]***  indirect effect = -0.11 [-0.19, -0.03]/ *K*^2^ = 0.32 |  |
| Johnson & O’Brien, 2013 | Self-Compassion Scale | Rumination Self-esteem [Rumination Questionnaire; Self-Esteem Scale]/  Rumination: *r* = -0.52***  Self-esteem: *r* = 0.60*** | Depression [Beck Depression Inventory]/  *r* = -0.49*** | Rumination: *r* = 0.38***  Self-esteem: *r* = -0.62*** | 335 students | Cross-sectional | PROCESS/Paralleled mediation model | ***Depression [outcome]***  Rumination: indirect effect = -0.0037 [-0.0068, -0.0011]/*PM* = 0.13 Self-esteem: indirect effect = -0.0104 [-0.0160, -0.0058]/*PM* = 0.36 |  |
| Hodgetts et al., 2021 | Self-Compassion Scale | Rumination [Ruminative Thought Style Questionnaire]/  *r* = -0.48*** | Depression [Epidemiologic Studies Depression Scale]/  *r* = -0.43*** | *r* = 0.48*** | 241 elders | Cross-sectional | PROCESS/Moderated mediation model | ***Depression [outcome]***  Rumination (men): indirect effect = -1.20 [-2.88, -0.003] (Unstandardized)/ *PM* = 0.14 Rumination (women): indirect effect = -5.03 [-7.45, -2.78] (Unstandardized)/*PM* = 0.58 |  |
| Fresnics & Borders, 2017 | Self-Compassion Scale | Rumination [Anger Rumination Scale]/  *r* = -0.50** | Anger [State Trait Anger Expression Inventory 2]/  *r* = -0.16* | r = 0.33** | 201 participants | Cross-sectional | PROCESS/Simple mediation model | ***Anger [outcome]***  Rumination: indirect effect = -0.02 [-0.01, -0.04]/*PM* = 0.13 |  |
| Jansen, Hoja, et al., 2021 | Self-Compassion Scale | Rumination Worry [Rumination-reflection questionnaire; Penn-State worry questionnaire]  Self-compassion[positive] – Rumination: *r* = -0.24**  Self-compassion[positive] – Worry: *r* = -0.20**  Self-compassion[negative] – Rumination: *r* = 0.57**  Self-compassion[negative] – Worry: *r* = 0.56** | Anxiety [Competition anxiety inventory]/  Self-compassion [positive]: *r* = -0.16*  Self-compassion [negative]: *r* = 0.38** | Rumination: *r* = 0.30**  Worry: *r* = 0.36** | 293 athletes | Cross-sectional | PROCESS/Paralleled mediation model | ***[Negative Self-compassion as independent variable]*** ***Somatic anxiety [outcome]*** Worry: indirect effect = 0.55 [0.14, 0.99]/*PM* = 0.30 Rumination: indirect effect = 0.05 [-0.37, 0.46]/PM = 0.03 |  |
| Casali et al., 2022 | Self-Compassion Scale | Worry [Penn State Worry Questionnaire]/  *r* = -0.37*** | Anxiety [Competition anxiety inventory]/  Somatic anxiety: *r* = -0.23**  Anxiety concern: *r* = -0.38** | Somatic anxiety: *r* = 0.31***  Anxiety concern: *r* = 0.39*** | 263 athletes | Cross-sectional | lavaan package in R/Mediation model with two outcomes | ***Somatic anxiety [outcome]*** Worry: indirect effect = -.11, *p* = .001/Not applicable ***Anxiety concern [outcome]*** Worry: indirect effect = -0.13, *p* < .001/Not applicable |  |
| Krieger et al., 2013 | Self-Compassion Scale | Rumination Experiential Avoidance [Response Styles Questionnaire; Cognitive-Behavioral Avoidance Scale]/  Symptom-focused rumination: *r* = -0.19*  Self-focused rumination: *r* = -0.16  Experiential avoidance: *r* = -0.30** | Depression [Beck Depression Inventory-II]/  *r* = -0.23** | Symptom-focused rumination: *r* = 0.40**  Self-focused rumination: *r* = 0.17*  Experiential avoidance: *r* = 0.36** | 142 clinically depressed patients; 196 never-depressed subjects | Cross-sectional | PROCESS/Simple mediation model | ***Depression [outcome]***  Symptom-focused rumination: indirect = -0.06 [-0.12, -0.002]/*PM* = 0.26  Self-focused rumination: indirect = -0.02 [-0.07, 0.002]/*PM* = 0.09  Experiential avoidance: indirect effect = -0.08 [-0.17, -0.02]/*PM* = 0.35 |  |
| Wadsworth et al., 2018 | Self-Compassion Scale-Short Form | Repetitive negative thoughts [Perseverative Thinking Questionnaire]/  Self-compassion[positive]: *r* = -0.38***  Self-compassion[negative]: *r* = 0.48*** | Depression Anxiety [Center for the Epidemiological Studies of Depression‑10; 7‑item Generalized Anxiety Disorder Scale]/  Self-compassion[positive] - Depression: *r* = -0.22***  Self-compassion[positive] - Anxiety: *r* = -0.22***  Self-compassion[negative] - Depression: *r* = 0.31***  Self-compassion[negative] - Anxiety: *r* = 0.30*** | Depression: *r* = 0.41***  Anxiety: *r* = 0.42*** | 582 adults with acute symptoms of psychopathology | Intervention | Mplus/Main path model | ***Negative self-compassion [independent variable] and Depression [outcome]***  Repetitive Negative thinking: indirect effect = 0.06 [0.03, 0.10]/Not applicable ***Negative self-compassion [independent variable] and Anxiety [outcome]***  Repetitive Negative thinking: indirect effect = 0.07 [0.04, 0.12]/ Not applicable  ***Positive self-compassion [independent variable] and Depression [outcome]***  Repetitive Negative thinking: indirect effect = -0.03 [-0.07, -0.004]/Not applicable  ***Positive self-compassion [independent variable] and Anxiety [outcome]***  Repetitive Negative thinking: indirect effect = -0.04 [-0.08, -0.01]/ Not applicable |  |
| Zhu et al., 2022 | Self-Compassion Scale-Short Form | Rumination: *r* = -0.18**  Catastrophizing: *r* = -0.32**  [Cognitive Emotion Regulation Questionnaire] | Fear of cancer recurrence [Fear of Cancer Recurrence Inventory‐Short Form] *r* = -0.26** | Rumination: *r* = 0.32**  Catastrophizing: *r* = 0.42** | 304 women with breast cancer | Cross-sectional | Parallelled mediation analysis | ***Fear of cancer revurrence [outcome]***  Rumination: Indirect effect = -0.022 [-0.07, 0.00]/*PM* = 0.07  Catastrophizing: Indirect effect = -0.096 [-0.17, -0.03]/*PM* = 0.31 |  |
| Karataş & Tüccar, 2025 | Self-Compassion Scale-Short Form | Rumination [Ruminative Response Scale-Short Form] | Stress [Secondary Traumatic Stress Scale] | Not applicable | 153 professionals | Cross-sectional | Mediation analysis | ***Stress [outcome]***  Indirect effect = -0.175 [-0.302, -0.033]/*PM* = 0.46 |  |
| Cabaços et al., 2023 | Self-Compassion Scale-Short Form | Repetitive negative thinking [Perseverative Thinking Questionnaire] *r* = -0.58* | Burnout [The Maslach Burnout Inventory-Student Survey] *r* = -0.37* | *r* = 0.50* | 202 medical and dentistry students | Longitudinal | Serial mediation model | ***Burnout [outcome]***  Indirect effect = 0.035 [0.0014, 0.0874]/Not applicable |  |
| Maddock, 2024b | Self-Compassion Scale-Short Form | Worry [The Penn State Worry Questionnaire]  Rumination [The Rumination Reflection Questionnaire] | Stress [The Perceived Stress Scale] | Not applicable | 83 social workers | Intervention | SEM (change scores) | ***Stress [outcome]***  Worry: Indirect effect = 0.12 [0.04, 0.23]; *PM* = 0.92  Rumination: Indirect effect = 0.08 [0.01, 0.18]/*PM* = 0.38 |  |
| Maddock, 2024a, 2025 | Self-Compassion Scale-Short Form | Worry [The Penn State Worry Questionnaire]  Rumination [The Rumination Reflection Questionnaire] | Stress [The Perceived Stress Scale]  Depression/Anxiety [The Hospital Anxiety and Depression Scale]  Well-being [Warwick-Edinburgh Mental Well-being Scale] | Not applicable | 121 social workers | Cross-sectional | SEM | ***Stress [outcome]***  Worry: Indirect effect = -0.29 [-0.41, -0.16]/*PM* = 0.88  Rumination: Indirect effect = -0.21 [-0.35, -0.08]/*PM* = 0.64  ***Anxiety [outcome]***  Worry: Indirect effect = -0.12 [-0.18, -0.06]/*PM* = 0.63  Rumination: Indirect effect = -0.16 [-0.24, -0.08]/*PM* = 0.80  ***Depression [outcome]***  Worry: Indirect effect = -0.05 [-0.10, 0.01]/*PM* = 0.55  Rumination: Indirect effect = -0.12 [-0.19, -0.05]/*PM* = 0.29  ***Well-being [outcome]***  Worry: Indirect effect = 0.04 [-0.06, 0.14]/*PM* = 0.12  Rumination: Indirect effect = 0.21 [0.07, 0.37]/*PM* = 0.62 |  |
| Arimitsu & Hofmann, 2015 | Self-Compassion Scale | Positive/negative automatic thoughts [Positive Automatic Thought Scale; Depression Anxiety Cognition Scale]  Positive automatic thoughts: *r* = 0.62*  Negative automatic thoughts: *r* = -0.58* | Depression Anxiety Life satisfaction [Beck Depression Inventory-II; Spielberger Trait Anxiety Inventory; Satisfaction with Life Scale]:  Depression: *r* = -0.45* Anxiety: *r* = -0.72* Life satisfaction: r = 0.50* | Positive automatic thoughts - Depression: *r* = -0.53*  Negative automatic thoughts - Depression: *r* = 0.58*  Positive automatic thoughts - Anxiety: *r* = -0.71*  Negative automatic thoughts - Anxiety: *r* = 0.64*  Positive automatic thoughts - Life satisfaction: *r* = 0.59*  Negative automatic thoughts - Life satisfaction: *r* = 0.43* | 233 students | Study 2: Cross-sectional | AMOS/SEM | ***Anxiety/Depression [outcome]***  Positive/Negative automatic thoughts: indirect effects are significant [parameters undisclosed]  ***Life satisfaction [outcome]*** Positive automatic thoughts: indirect effect is significant [parameters undisclosed]  Negative automatic thoughts: indirect effect is not significant [parameters undisclosed] |  |
| Norman et al., 2020 | Self-Compassion Scale | Belief about voice [Belief about Voices Questionnaire‐Revised]/  *r* = -0.44** | Distress [Psychotic Symptom Rating Scale]/  *r* = -0.40** | *r* = 0.48** | 53 help‐seeking voice hearers | Cross-sectional | PROCESS/Simple mediation model | ***Voice distress [outcome]***  Beliefs about voice: indirect effect is significant [parameters undisclosed] |  |
| Yela et al., 2022 | Self-Compassion Scale | Experiential avoidance [Acceptance and Action Questionnaire]/Not applicable | Anxiety Depression Well-being [Hospital Anxiety and Depression Scale; Psychological Wellbeing Scales] /Not applicable | Not applicable | 50 participants | Intervention | PROCESS/Simple mediation model | ***Anxiety [outcome]***  Experiential avoidance: indirect effect = -0.26 [-0.51, -0.04]/*PM* = 0.41 ***Depression [outcome]***  Experiential avoidance: indirect effect = -0.34 [-0.59, -0.12]/ *PM* = 0.54 ***Well-being [outcome]***  Experiential avoidance: indirect effect = 0.17 [0.01, 0.37]/*PM* = 0.20 |  |
| Adie et al., 2021 | Self-Compassion Scale | Avoidance [Behavioral Activation for Depression Scale]/  *r* = -0.62*** | Depression [Depression Anxiety and Stress Scale‑21] /  *r* = -0.49 | *r* = 0.63** | 242 participants | Cross-sectional | Mplus/SEM | ***Depression [outcome]***  Avoidance: indirect effect = -0.33, [-0.39, -0.26]/*PM* = 0.67 |  |
| Bates et al., 2021 | Self-Compassion Scale-Short Form | Expressive suppression Cognitive reappraisal [Emotion Regulation Questionnaire]/  Expressive suppression: *r* = 0.54*** Cognitive reappraisal: *r* = -0.17*** | Anxiety [Social Phobia Scale; Social Interaction Anxiety Scale]/  Social anxiety: *r* = -0.52***  Social phobia: *r* = -0.45*** | Expressive suppression – Social anxiety: *r* = 0.31***  Cognitive reappraisal – Social anxiety: *r* = -0.33***  Expressive suppression – Social phobia: *r* = 0.23***  Cognitive reappraisal – Social phobia: *r* = -0.29*** | 750 students | Cross-sectional | Mplus/SEM | ***Social anxiety [outcome]*** Expressive suppression: indirect effect = -0.05, p < 0.001/*PM* = 0.09 Cognitive reappraisal: indirect effect = -0.03, p > 0.05/*PM* = 0.05 ***Social phobia [outcome]*** Expressive suppression: indirect effect = -0.04, p < 0.01/*PM* = 0.08 Cognitive reappraisal: indirect effect = -0.04, p > 0.05/*PM* = 0.08 |  |
| McBride et al., 2022 | Self-Compassion Scale | Cognitive reappraisal: *r* = 0.61**  Expressive suppression: *r* = -0.27** [The Emotion Regulation Questionnaire] | Social anxiety [The Social Interaction Anxiety Scale]  *r* = -0.60** | Cognitive reappraisal: *r* = -0.48**  Expressive suppression: *r* = 0.37** | 363 participants [33 people with SAD] | Cross-sectional | Mediation analysis | ***Anxiety [outcome]***  Cognitive reappraisal: Indirect effect = -0.06/*PM* = 0.16  Expressive suppression: Indirect effect = -0.04/*PM* = 0.11 |  |
| Ewert et al., 2018 | Self-Compassion Scale | Denial [Carver's Brief COPE-Inventory]/  *r* = -0.305 | Shame [Positive and Negative Affect Schedule]/  *r* = -0.373*** | *r* = 0.62** | 105 participants | Experimental Study | Baron and Kenny's causal step approach | ***Shame [outcome]***  Denial: indirect effect z = 1.98, *p* < 0.05/Not applicable |  |
| Kreemers et al., 2020 | Self-Compassion Scale | Self-criticism [Self-Criticism Questionnaire]/  *r* = -0.44** | Affect [Positive and Negative Affect Schedule]/  Negative activating affect: *r* = -0.44**  Negative deactivating affect: *r* = -0.52**  Positive activating affect: *r* = 0.48**  Positive deactivating affect: *r* = 0.48** | Negative activating affect: *r* = 0.58**  Negative deactivating affect: *r* = 0.60**  Positive activating affect: *r* = -0.35**  Positive deactivating affect: *r* = -0.45** | 180 participants | Experimental study | PROCESS/Serial mediation model | ***[Serial mediation model]***  Condition - Self-compassion - Self-criticism - Negative activating affect: Indirect effect = -0.10 [-0.225, -0.007]/*PM* = 0.23 Condition - Self-compassion - Self-criticism - Negative deactivating affect: Indirect effect = -0.09 [-0.205, -0.009]/*PM* = 0.31 Condition - Self-compassion - Self-criticism - Positive activating affect: Indirect effect = 0.03 [-0.001, -0.093]/*PM* = 0.68 Condition - Self-compassion - Self-criticism - Negative deactivating affect: Indirect effect = -0.06 [0.004, 0.138]/*PM* = 0.29 |  |
| Angus & Phillips, 2021 | Self-Compassion Scale | Counterfactual thinking [Counterfactual Thinking Negative Events Scale]/  *r* = -0.33*** | Depression [Depression, Anxiety and Stress Scales – Short Form]/  *r* = -0.51*** | *r* = 0.36*** | 167 students | Cross-sectional | PROCESS/Paralleled mediation model | ***Depression [outcome]***  Counterfactual thinking: Indirect effect = -0.06 [-0.14, 0.03]/*PM* = 0.16 |  |
| Vidal et al., 2024 | Self-Compassion Scale-Short Form | Preservation of negative emotion [Emotional Reactivity Intensity and Perseveration  Scale] *r* = -0.57*  Emotional Overproduction [Emotional Overproduction Scale] *r* = -0.67* | Depression [Brief Symptom Inventory-18] *r* = -0.60* | Preservation of negative emotion: *r* = 0.50*  Emotional Overproduction: *r* = 0.61* | 453 participants | Cross-sectional | SEM | ***Depression [outcome]***  Preservation of negative emotion: Indirect effect = -0.046 [-0.084, -0.008]/Not applicable  Emotional Overproduction: Indirect effect = -0.120 [-0.167, -0.074]/Not applicable |  |
| Makadi & Koszycki, 2020 | Self-Compassion Scale-Short Form | Mindfulness [Five-Facet Mindfulness Questionnaire]  Awareness: *r* = 0.44** Describe: *r* = 0.22* Nonjudge: *r* = 0.41** Non-react: *r* = 0.56** Observe: *r* = 0.24* | Anxiety [Liebowitz Social Anxiety Scale; Social Phobia Inventory]/  Social anxiety: *r* = -0.42**  Social phobia: *r* = -0.43** | Awareness - Social anxiety: *r* = -0.23* Describe - Social anxiety: *r* = -0.31** Nonjudge - Social anxiety: *r* = -0.20* Non-react - Social anxiety: *r* = -0.22* Observe - Social anxiety: *r* = -0.11  Awareness - Social phobia: *r* = -0.29** Describe - Social phobia: *r* = -0.25* Nonjudge - Social phobia: *r* = -0.23* Non-react - Social phobia: *r* = -0.25* Observe - Social phobia: *r* = -0.09 | 136 outpatients who met DSM-5 criteria for SAD | Cross-sectional | PROCESS/Paralleled mediation model | ***Social Anxiety [outcome]*** Awareness: indirect effect = 0.01 [-0.18, 0.18]/*PM* = 0.01 Describe: indirect effect = -0.15 [-0.34, -0.02]/*PM* = 0.15 Nonjudge: indirect effect = -0.04 [-0.23, 0.13]/*PM* = 0.04 Non-react: indirect effect = 0.05 [-0.22, 0.35]/*PM* = 0.05 Observe: indirect effect = 0.05 [-0.04, 0.19]/*PM* = 0.05 ***Social Phobia [outcome]*** Awareness: indirect effect = -0.04 [-0.14, 0.07]/*PM* = 0.07 Describe: indirect effect = -0.15 [-0.13, -0.00]/*PM* = 0.27 Nonjudge: indirect effect = -0.02 [-0.11, 0.06]/*PM* = 0.04 Non-react: indirect effect = 0.00 [-0.16, 0.17]/*PM* = 0 Observe: indirect effect = 0.03 [-0.02, 0.10]/*PM* = 0.05 |  |
| Townshend & Caltabiano, 2019 | Self-Compassion Scale | Mindfulness [Five-Facet Mindfulness Questionnaire]/  *r* = 0.71*** | Depression [Edinburgh Postnatal Depression Scale]/  *r* = -0.45*** | *r* = -0.51*** | 77 partnered pregnant women | Longitudinal | PROCESS/Serial mediation model | ***[serial mediation model]***  Perinatal depression T1 - Self-compassion T1 - Perinatal depression T2: indirect effect = -0.06 [0.02, 0.16]/*PM* = 0.11  Perinatal depression T1 - Self-compassion T1 - Mindfulness T2 - Perinatal depression T2: indirect effect = 0.06 [0.01, 0.15]/*PM* = 0.11 |  |
| Hsieh et al., 2021 | Compassionate Engagement and Action Scales | Mindfulness [Five-Facet Mindfulness Questionnaire]/  *r* = 0.50*** | Depression [Beck Depression Inventory-II]/  *r* = -0.31*** | *r* = -0.55*** | 123 family caregivers | Longitudinal | PROCESS/Serial mediation model | ***[Serial mediation model]***  Self-compassion T1 - Mindfulness T2 - Caregiver Stress T3 - Depressive symptoms T4: indirect effect = - 0.079 [-0.171, -0.014]/ Not applicable |  |
| Gouveia et al., 2016 | Self-Compassion Scale | Mindfulness [Interpersonal Mindfulness in Parenting Scale]/  *r* = 0.54** | Stress [Parenting Stress Index–Short Form]/  *r* = -0.57** | *r* = -0.43** | 333 parents | Cross-sectional | PROCESS/Simple mediation model | ***Parenting stress [outcome]***  Mindfulness parenting: Indirect effect = -1.30 [-2.339 -0.303] (Unstandardized)/Not applicable |  |
| Carvalho et al., 2018 | Self-Compassion Scale-Short Form | Acceptance [Chronic Pain Acceptance Questionnaire‐8]/  Activity engagement: *r* = 0.40***  Pain willingness: *r* = -0.10 | Depression [Depression, Anxiety, and Stress Scale‐21]/ *r* = -0.55*** | Activity engagement: *r* = -0.43***  Pain willingness: *r* = -0.12 | 231 women with chronic pain | Cross-sectional | AMOS/Path analysis | ***Depression [outcome]***  Activity engagement: indirect effect = -0.089 [-0.151, -0.043]/Not applicable  Pain willingness: indirect effect is not significant [parameters undisclosed] |  |
| Stephenson et al., 2018 | Self-Compassion Scale-Short Form | Self-worth (In)Tolerance [Self-Worth Scale; Survey of Personal Beliefs] | Depression Anxiety [Costello and Comrey Depression and Anxiety Scales] | Not applicable | 184 students | Cross-sectional | PROCESS/Paralleled mediation model | ***Depression [outcome]*** Self-worth: indirect effect = 0.10 [0.03, 0.19]; Tolerance: indirect effect = -0.01 [-0.06, 0.02]; ***Anxiety [outcome]*** Self-worth: indirect effect = -0.05 [-0.13, 0.01]; Tolerance: indirect effect = -0.04 [-0.11, -0.01] |  |
| Kaçar-Başaran, 2025; Kaçar-Başaran & Gökdağ, 2025 | Self-Compassion Scale-Short Form | Intolerance [Intolerance of Uncertainty Scale-Short Form] | Distress [Depression Anxiety Stress Scales–21]  OCD symptoms [Padua Inventory-Washington State University Revision] | Not applicable | 400 adults | Cross-sectional | SEM | ***Distress [outcome]***  Indirect effect = -0.12 [-0.19, -0.07]  OCD  Indirect effect = -0.48 [-0.66, -0.34] |  |
| Morgenroth et al., 2022 | Self-Compassion Scale | Decentering [Experience Questionnaire]/  Distanced perspective: *r* = 0.48**  Accepting self-perception: *r* = 0.51** | Depression Anxiety [Hospital Anxiety and Depression Scale]/  Depression: *r* = -0.34*  Anxiety: *r* = -0.28 | Distanced perspective - Depression: *r* = -0.34*  Accepting self-perception - Depression: *r* = -0.43**  Distanced perspective - Anxiety: *r* = -0.52**  Accepting self-perception - Anxiety: *r* = -0.27 | 45 left ventricular assist device patients | Cross-sectional | PROCESS/Paralleled mediation model | ***Anxiety [outcome]***  Decentering: indirect effect = -2.00 [-3.82, -0.37]/*PM* = 0.93; ***Depression [outcome]***  Decentering: indirect effect = -1.70 [-3.44, -0.10]/*PM* = 0.65. |  |
| Phillips, 2018 | Self-Compassion Scale | Balanced time perspective Optimism Savoring-anticipating [short form Zimbardo Time Perspective Inventory; Life Orientation Test-Revised; Savoring Beliefs Inventory]/  Balanced time perspective: *r* = -0.56*** Optimism: *r* = 0.80*** Savoring-anticipating: r = 0.41*** | Depression Life satisfaction [Depression and Anxiety Stress Scales; Satisfaction With Life Scale]/  Depression: *r* = -0.67*** Life satisfaction: *r* = 0.58*** | Balanced time perspective - Depression: *r* = 0.65***  Optimism - Depression: *r* = -0.70*** Savoring-anticipating - Depression: *r* = -0.52***  Balanced time perspective - Life satisfaction: *r* = -0.62***  Optimism - Life satisfaction: *r* = 0.60*** Savoring-anticipating - Life satisfaction: *r* = 0.47*** | 157 participants | Cross-sectional | PROCESS/Paralleled mediation model | ***Depression [outcome]*** Balanced time perspective: indirect effect = -0.17 [-0.26, -0.09]/*PM* = 0.27; Optimism: indirect effect = -0.12 [-0.29, 0.07]/*PM* = 0.19; Savoring-anticipating: indirect effect = -0.06 [-0.12, -0.002]/*PM* = 0.10 ***Life satisfaction [outcome]*** Balanced time perspective: indirect effect = 0.18 [0.08, 0.30]/*PM* = 0.30; Optimism: indirect effect = 0.09 [-0.07, 0.25]/*PM* = 0.15; Savoring-anticipating: indirect effect = 0.05 [-0.002, 0.12]/*PM* = 0.08 |  |
| Shi et al., 2025 | Self-Compassion Scale-Short Form | Self-esteem [Rosenberg Self-Esteem scale] | Depression | Not applicable | 4312 students | Longitudinal | Random intercept cross-lagged panel model | ***Depression [outcome]***  Indirect effect = -0.003 [-0.005, -0.001];  -0.003 [-0.005, -0.001];  -0.002 [-0.004, -0.001]; |  |
| Eccles et al., 2023 | Self-Compassion Scale | Stigma [Stigma Scale for Chronic Illness]/  *r* = -0.56*** | Depression Anxiety Stress [Depression, Anxiety, and Stress Scale]/  Depression: *r* = -0.73***  Anxiety: *r* = -0.48***  Stress: *r* = -0.65*** | Depression: *r* = 0.64***  Anxiety: *r* = 0.58***  Stress: *r* = 0.63*** | 153 individuals | Cross-sectional | PROCESS/Simple mediation model | ***Depression [outcome]***  Stigma: indirect effect = -1.494 [-2.302, -0.794]/*PM* = 0.27; ***Anxiety [outcome]***  Stigma: indirect effect = -1.391 [-2.067, -0.826]/*PM* = 0.59; ***Stress [outcome]***  Stigma: indirect effect = -1.338 [-2.172, -0.636]/*PM* = 0.28 |  |
| Coutts et al., 2023 | Self-Compassion Scale-Short Form | Self-concept clarity [The Self-Concept Clarity Scale] *r* = 0.50** | Depression [m CES-D] *r* = -0.52**  Stress [Perceived Stress Scale] *r* = -0.61**  Life satisfaction [Satisfaction with Life Scale] *r* = 0.46** | Depression: *r* = -0.57**  Stress: *r* = -0.64**  Life satisfaction: *r* = 0.37** | 253 undergraduate students | Cross-sectional | Mediation analysis | ***Depression [outcome]*** Indirect effect = -0.21 [-0.283, -0.146]/*PM* = 0.39  ***Stress [outcome]***  Indirect effect = -0.19 [-0.263, -0.117]/*PM* = 0.30  ***Life satisfaction [outcome]***: Indirect effect = 0.09 [0.009, 0.174]/*PM* = 0.19 |  |
| Ghorbani et al., 2012 | Self-Compassion Scale | Integrative self-knowledge [Integrative Self-Knowledge Scale]/  *r* = 0.38*** | Depression Anxiety [Costello and Comrey Depression Scale]/  Depression: *r* = -0.36***  Anxiety: *r* = -0.24*** | Depression: *r* = -0.46***  Anxiety: *r* = -0.42*** | 238 Muslims | Cross-sectional | PROCESS/Moderated mediation model | ***Depression [outcome]*** Integrative self-knowledge : indirect effect = -0.215 [-0.316, -0.123]/Not applicable ***Anxiety [outcome]***  Integrative self-knowledge: indirect effect = -0.186 [-0.278, -0.107]/Not applicable |  |
| Brown-Beresford & mclaren, 2022 | | Self-Compassion Scale | Internalized heterosexism [Lesbian Internalized Homophobia Scale]/  *r* = -0.24*** | Depression [Center for Epidemiological Studies – Depression Scale]/  *r* = -0.61*** | *r* = 0.26*** | 498 bisexual women and 416 lesbian women | Cross-sectional | PROCESS/Moderated mediation model | ***Depression [outcome]***  Internalized heterosexism (Lesbian): indirect effect = -0.66, [-1.08, -0.31]/*PM* = 0.07;  Internalized heterosexism (bisexual women): indirect effect = -0.32 [-0.66, -0.04]/*PM* = 0.03 |
| Ristvej et al., 2024 | Self-Compassion Scale | Internalized heterosexism [The Revised Internalized Homophobia Scale] | Depression [The Center for Epidemiologic Studies Depression Scale] |  | 1285 gay men and 487 bisexual men | Cross-sectional | Multiple linear regression analysis | Self-warm: Indirect effect = -0.52 [-0.74, -0.33]  Self-coldness: Indirect effect = 0.33 [0.17, 0.51] |  |
| Zhu et al., 2020 | Self-Compassion Scale-Short Form | Illness perception [Illness Perception Questionnaire Revised]/  Personal control: *r* = 0.22** Treatment control: *r* = 0.21** Consequence: *r* = -0.31** Timeline cyclical: *r* = -0.18** | Depression Anxiety [Patient Health Questionnaire; State-Trait Anxiety Inventory]/  Depression: *r* = -0.37**  Anxiety: *r* = - 0.39** | Consequence - Depression: *r* = 0.42**  Personal control - Depression: *r* = -0.19** Treatment control - Depression: *r* = -0.24** Timeline cyclical - Depression: *r* = 0.38**  Consequence - Anxiety: *r* = 0.35**  Personal control - Anxiety: *r* = -0.26** Treatment control - Anxiety: *r* = -0.24** Timeline cyclical - Anxiety: *r* = 0.20** | 301 cancer patients | Cross-sectional | PROCESS/Paralleled mediation model | ***Depression [outcome]*** Personal control: indirect effect = -0.014 [-0.05, 0.01]/Not applicable; Treatment control: indirect effect = -0.006 [-0.03, 0.01]/Not applicable; Consequence: indirect effect = -0.064 [-0.11, -0.02]/*K*^2^ = 0.074; Timeline cyclical: indirect effect-0.037 [-0.08, -0.01] *K*^2^ = 0.043. ***Anxiety [outcome]*** Personal control: indirect effect = -0.023 [-0.05, -0.01]/ *K*^2^ = 0.044; Treatment control: indirect effect = -0.007 [-0.03, 0.01]/Not applicable; Consequence: indirect effect = -0.039 [-0.07, -0.01]/ *K*^2^ = 0.072; Timeline cyclical: indirect effect = 0.001 [-0.02, 0.02]/Not applicable. |  |
| Zhou et al., 2013 | Self-Compassion Scale | Cognitive style [Cognitive Style Questionnaire]/Not applicable | Hopelessness depression [Hopelessness Depression Symptom Questionnaire] /Not applicable | /Not applicable | 418 students | Cross-sectional | AMOS/SEM | Significant but not report explicit parameters/Not applicable |  |
| Gerber et al., 2021 | Self-Compassion Scale | Autonomy  Relatedness  Competence [Basic Psychological Needs Scale]/  Autonomy: *r* = 0.40**  Relatedness: *r* = 0.10  Competence: *r* = 0.44** | Burnout [Parental Burnout Inventory]/  *r* = -0.38** | Autonomy: *r* = -0.43**  Relatedness: *r* = -0.47**  Competence: *r* = -0.33** | 91 parents | Cross-sectional | Two-step hierarchical regression analysis | Not significant, did not report explicit parameters |  |
| Gerber & Anaki, 2021 | Self-Compassion Scale | Autonomy Relatedness [Psychological Needs Scale]/  Autonomy: *r* = 0.45**  Relatedness: *r* = 0.40**  Competence: *r* = 0.14 | Burnout [Maslach Burnout Inventory]/  *r* = -0.34** | Autonomy: *r* = -0.50**  Relatedness: *r* = -0.40**  Competence: *r* = -0.42** | 109 professional caregivers | Cross-sectional | Partial Least Squares (PLS) SEM path modeling | ***Burnout [outcome]***  Autonomy: indirect effect = -0.13 [-0.26, -0.03]/Not applicable; Relatedness: indirect effect parameter not reported [-0.10, 0.01]/Not applicable;  Competence: indirect effect parameter not reported [-0.13, 0.02]/Not applicable. |  |
| L. Brown et al., 2016 | Self-Compassion Scale | Attitudes to ageing [Attitudes to ageing questionnaire]/  Psychosocial loss: *r* = -0.69**  Physical change: *r* = 0.78**  Psychological growth: *r* = -0.78** | Depression Well-being [Centre for epidemiological studies depression scale; Warwick-Edinburgh mental well-being]/  Depression: *r* = 0.16*  Well-being: *r* = 0.03 | Psychosocial loss - Depression: *r* = 0.63  Physical change - Depression: *r* = -0.57  Psychological growth - Depression: *r* = -0.51**  Psychosocial loss – Well-being: *r* = -0.64  Physical change - Well-being: *r* = 0.64**  Psychological growth - Well-being: *r* = 0.54** | 7615 adults | Cross-sectional | Mplus/SEM | ***[self-compassion subscales as independent variables]***  Positive self-compassion - Physical change - Depression: indirect effect = -0.078, p = 0.007/Not applicable; Negative self-compassion - Physical change - Depression: indirect effect = 0.089, p = 0.002/Not applicable;  Positive self-compassion – Psychosocial loss - Depression: indirect effect = -0.058, p = 0.002/Not applicable;  Negative self-compassion – Psychosocial loss - Depression: indirect effect = 0.13, p = 0.012/Not applicable; Positive self-compassion - Physical change - Well-being: indirect effect = 0.12, p = 0.001/Not applicable; Negative self-compassion - Physical change - Well-being: indirect effect = -0.13, p < 0.001/Not applicable |  |
| Pyszkowska et al., 2024 | Self-Compassion Scale-Short Form | Time perspective [Zimbardo Time Perspective Inventor] *r* = -0.41** | Depression [Depression, Anxiety and Stress Scale in the 21-item version] *r* = -0.43** | *r* = 0.52** | 300 participants with or without depression | Cross-sectional | SEM | Not significant |  |
| Ying, 2009 | Self-Compassion Scale | Sense of coherence [Antonovsky's 13-item Sense of Coherence Questionnaire.]/Not applicable | Depression [California Psychological Inventory-Depression Scale] /Not applicable | Not applicable | 65 students | Cross-sectional | Sobel's test | ***Depression [outcome]***  Sense of coherence: indirect effect *z* = 2.18, *p* = 0.03 |  |
| Xie et al., 2023 | Self-Compassion Scale | Rejection Sensitivity [Rejection Sensitivity Questionnaire] *r* = -0.38** | Loneliness [The 20-item UCLA Loneliness Scale] *r* = -0.46** | *r* = 0.57** | 275 participants | Cross-sectional | Mediation analysis | ***Loneliness [outcome]***  Indirect effect = -0.179 [-0.248, -0.112]/*PM* = 0.39 |  |
| Allen et al., 2024 | Self-Compassion Scale | Perceived Social Support [Multidimensional Scale of Perceived Social Support] | PTSS [The PTSD Checklist] |  | 261 participants  living in four rural North Carolina | Longitudinal | Mediation analyses | ***PTSS [outcome]***  Indirect effect = -0.66 [-1.19, -0.31]/*PM* = 0.35 |  |
| Blankenship & Hogge, 2024 | Self-Compassion Scale | Emotion regulation difficulty [The Difficulties in Emotion Regulation Scale] *r* = 0.61** | Well-being [The Psychological Well-Being] *r* = 0.64** | *r* = -0.73** | 335 adults who reported experiencing CSA prior to the age of 14 | Cross-sectional | Mediation analysis | ***Well-being [outcome]***  Indirect effect = 17.10 [11.62, 22.98]; |  |
| Julian et al., 2025 | Self-Compassion Scale | Emotion regulation difficulty [The Difficulties in Emotion Regulation Scale] *r* = -0.70** | Well-being [The Scales of Psychological Wellbeing] *r* = 0.59** | *r* = -0.69** | 748 undergraduates | Cross-sectional | Mediation analysis | ***Well-being [outcome]***  Indirect effect = -0.17 [-0.24, -0.12]/Not applicable |  |
| Li et al., 2021 | Self-Compassion Scale | Positive coping [Simplified Coping Style Questionnaire]/  *r* = 0.29*** | Life satisfaction [Satisfaction with Life Scale]/  *r* = 0.43*** | *r* = 0.21*** | 337 adults | Cross-sectional | PROCESS/Moderated mediation model | ***Life satisfaction [outcome]***  Coping (men): indirect effect = 0.15 [0.05, 0.24]/Not applicable; Coping (women): indirect effect = -0.07 [-0.15, 0.02] /Not applicable; |  |
| Ewert et al., 2024 (Study 1) | Self-Compassion Scale | Coping  [Brief COPE inventory]  Engagement coping: *r* = 0.47**  Disengagement coping: *r* = -0.34** | Affective Well‑being [Positive and Negative Affect Schedule] *r* = 0.42 | Engagement coping: *r* = 0.37**  Disengagement coping: *r* = -0.37** | 287 participants | Longitudinal design for 12 weeks | Cross lagged panel model | ***Well-being [outcome]***  Engagement coping: indirect effect = -0.001 [-0.003， 0.002]/Not applicable;  Disengagement coping: indirect effect = 0.001 [-0.002, 0.001]/Not applicable |  |
| Ewert et al., 2024 (Study 2) | Self-Compassion Scale | Coping  [Brief COPE inventory]  Engagement coping: *r* = 0.31**  Disengagement coping: *r* = -0.03 | Affective Well‑being [Positive and Negative Affect Schedule] *r* = 0.23** | Engagement coping: *r* = 0.25**  Disengagement coping: *r* = -0.31** | 2668 participants | Longitudinal design for 4 months | Cross lagged panel model | ***Well-being [outcome]***  Engagement coping: indirect effect = 0.013 [-0.001, 0.029] /Not applicable  Disengagement coping: indirect effect = 0.003 [-0.001, 0.009]/Not applicable |  |
| Voon et al., 2022 | Self-Compassion Scale | Resilience [Connor-Davidson Resilience Scale]/Not applicable | Well-being [Scale of Psychological Well-Being]/Not applicable | Not applicable | 408 counselors | Cross-sectional | Partial Least Square-Structural Equation Modeling | ***Well-being [outcome]***  Resilience: indirect effect = 0.234 [0.180, 0.288]/*PM* = 0.32 |  |
| Eghbali et al., 2022 | The Self-Compassion Scale Short Form | Resilience [Connor-Davidson Resilience Scale] *r* = 0.66** | Well-being [Ryff’s Psychological Well-Being Scales] *r* = 0.65** | *r* = 0.78** | 410 people with multiple sclerosis | Cross-sectional | SEM | ***Well-being [outcome]***  Indirect effect = 0.50 [0.43, 0.58]/*PM* = 0.70 |  |
| Bogerd et al., 2023 | The Self-Compassion Scale | Resilience [Brief Resilience Scale]  *r* = 0.24** | Professional Fulfilment [Professional Fulfilment Scale] *r* = 0.17** | *r* = 0.32** | 374 cardiologists | Cross-sectional | Mediation analysis | ***Fulfilment [outcome]***  Indirect effect = 0.049 [0.020, 0.086]/*PM* = 0.33 |  |
| Skinner & Kuijer, 2024 | Self-Compassion Scale-Short Form | Resilience [The Brief Resilience Scale] *r* = 0.53** | Quality of life [The EHP-30 disease-specific instrument] *r* = -0.40** | *r* = -0.29** | 603 participants with endometriosis | Cross-sectional | Mediation analysis | ***Quality of life [outcome]***  Indirect effect = -0.88 [-2.08, 0.32]/Not applicable |  |
| Shin, 2019 | Self-Compassion Scale | Worry [Employment Anxiety Scale]/  *r* = -0.33*** | Life satisfaction [Satisfaction with the Life Scale]/  *r* = 0.380*** | *r* = -0.32*** | 214 students | Cross-sectional | PROCESS/Simple mediation model | ***Life satisfaction [outcome]***  Worry: indirect effect = 0.031 [0.011, 0.052]/*PM* = 0.19 |  |
| Özönder Ünal et al., 2023 | Self-Compassion Scale-Short Form | Mindfulness [The Freiburg Mindfulness Inventory]  Experiential avoidance [The Acceptance and Action Questionnaire]  Cognitive Fusion [The Cognitive Fusion Questionnaire] | Posttraumatic Growth [The Posttraumatic Growth Inventory]  *r* = 0.61* | Mindfulness: *r* = 0.26*  Experiential avoidance: *r* = -0.43**  Cognitive Fusion: *r* = -0.45** | 253 patients with cancer diagnosis | Cross-sectional | Parallelled mediation analysis | ***PTG [outcome]***  Mindfulness: Indirect effect = 0.01 [0.00, 0.04]/*PM* = 0.03  Experiential avoidance: Indirect effect = 0.04 [0.01, 0.08]/*PM* = 0.08  Cognitive Fusion: Indirect effect = 0.05 [0.01, 0.12]/*PM* = 0.11 |  |
| Rehman et al., 2024 | Self-Compassion Scale- | Cognitive reappraisal/Expressive suppression [The Emotion Regulation Scale] Cognitive reappraisal: *r* = 0.27**  Expressive suppression: *r* = -0.22** | Mental health [General Health Questionnaire]  *r* = 0.35** | Cognitive reappraisal: *r* = 0.42***  Expressive suppression: *r* = -0.57*** | 405 academic teachers | Cross-sectional | Serial mediation analysis | ***Mental health [outcome]***  Cognitive reappraisal: Indirect effect = 0.037 [0.010, 0.078]/*PM* = 0.05  Expressive suppression: Indirect effect = -0.021 [-0.041, -0.002]/*PM* = 0.03 |  |
| Deniz, 2021 | | Self-Compassion Scale | Intolerance [Intolerance of Uncertainty Scale]/  *r* = -0.58*** | Well-being [Subjective Happiness Scale]/  *r* = 0.59*** | *r* = -0.43** | 667 people | Cross-sectional | PROCESS/Serial mediation model | ***[serial mediation model]***  Self-compassion - Intolerance - Well-being: indirect effect = 0.016 [0.004, 0.027]/*PM* = 0.11; Self-compassion - Fear of Covid - Well-being: indirect effect = 0.005 [0.002, 0.009]/*PM* = 0.03; Self-compassion - Intolerance - Fear of Covid - Well-being: indirect effect = 0.004 [0.001, 0.007]/*PM* = 0.03 |
| Munroe et al., 2022 | Self-Compassion Scale | Instrumental support Positive reframing [Brief COPE Inventory]  Instrumental support: *r* = 0.22* Positive reframing: *r* = 0.34** | Posttraumatic Growth [Posttraumatic Growth Inventory]/  *r* = 0.29** | Instrumental support: *r* = 0.49* Positive reframing: *r* = 0.55** | 111 adults | Cross-sectional | PROCESS/Paralleled mediation model | ***PTG [outcome]***  Active coping: indirect effect = 0.11 [0.03, 0.21]/*PM* = 0.38; Instrumental support: indirect effect = 0.05 [0.0003, 0.13]/*PM* = 0.17; Positive reframing: indirect effect = 0.09 [0.02, 0.17]/*PM* = 0.31 |  |
| Wong & Yeung, 2017 | Self-Compassion Scale (positive elements) | Acceptance Positive reframing [Brief COPE]/  Acceptance: *r* = 0.26** Positive reframing: *r* = 0.38**  Presence of meaning: *r* = 0.43** | Posttraumatic Growth [Posttraumatic Growth Inventory]/  *r* = 0.26** | Acceptance: *r* = 0.39** Positive reframing: *r* = 0.50**  Presence of meaning: *r* = 0.34** | 601 students | Cross-sectional | Mplus/SEM | ***PTG [outcome]***  Positive reframing: indirect effect = 0.19 [0.10, 0.28]/*PM* = 0.51;  Presence of meaning: indirect effect = 0.08 [0.03 0.14]/*PM* = 0.22 Acceptance: indirect effect = 0.05 [-0.01, 0.12]/PM = 0.14 |  |
| Zipagan & Galvez Tan, 2023 | Self -Compassion  Scale | Meaning of Life [Meaning in Life Questionnaire]  *r* = 0.58**  Self-acceptance [the Unconditional Self-Acceptance Questionnaire] *r* = 0.75** | Life satisfaction [the Satisfaction with Life Scale] *r* = 0.53** | Meaning of Life: *r* = 0.63**  Self-acceptance: *r* = 0.45** | 178 adults | Cross-sectional | Serial mediation analysis | ***Life satisfaction [outcome]***  Self-acceptance: indirect effect = 0.12 [−0.296, 0.425]/Not applicable  Meaning in life: indirect effect = 0.12 [0.271, 0.770], *PM* = 0.17. |  |
| Roxas et al., 2019 | Self-Compassion Scale | Forgiveness [Heartland Forgiveness Scale]/Not applicable | Well-being [Subjective Well-Being Scale for Filipinos- Short Form] /Not applicable | Not applicable | 231 counselors | Cross-sectional | Bootstrap procedure | ***Well-being [outcome]***  Forgiveness: indirect effect = 0.082 [0.052, 0.143] /Not applicable |  |
| Nguyen & Le, 2021 | Self-Compassion Scale | Gratitude [Gratitude Scale]/  *r* = 0.29** | Well-being [WHO-5 Well-Being Index]/  *r* = 0.59** | *r* = 0.38** | 509 adults | Cross-sectional | PROCESS/Paralleled mediation model | ***Well-being [outcome]***  Gratitude: indirect effect = 0.07 [0.04, 0.10]/*PM* = 0.12 |  |
| Salehi et al., 2023 | Self-Compassion Scale-Short Form | Self-efficacy [The pain self-efficacy questionnaire]  *r* = 0.66** | Quality of life [Quality of life questionnaire]  *r* = 0.63** | *r* = 0.65** | 298 elderly people with cardiovascular disease | Cross-sectional | Path analysis | ***Quality of life [outcome]***  Indirect effect = 0.16, p < 0.01/*PM* = 0.35 |  |
| Chong & Chan, 2023 | Self-Compassion Scale-Short Form | Internalized Homonegativity [Lesbian, Gay, and Bisexual Identity Scale]  Self-warm: *r* = -0.06;  Self-coldness: *r* = 0.21*** | Life satisfaction [Satisfaction With  Life Scale] Self-warm: *r* = 0.27***;  Self-coldness: *r* = -0.42*** | *r* = -0.24*** | 505 LGB adults | Cross-sectional | SEM | ***Life satisfaction [outcome]***  Self-warm: Indirect effect = 0.04 [0.01, 0.07]/Not applicable  Self-coldness: Indirect effect = -0.05 [-0.10, -0.02]/Not applicable |  |
| Lefebvre et al., 2021 | Self-Compassion Scale-Short Form | Social safeness [Social Safeness and Pleasure Scale]/  *r* = 0.33** | Flourishing [Flourishing Scale]/  *r* = 0.28** | *r* = 0.65** | 101 adults | Longitudinal | Mplus/Multilevel modelling | ***Flourishing [outcome]***  Social safeness: indirect effect = 0.23 [0.13, 0.33]/*PM* = 036 |  |
| Quang et al., 2022 | Self-Compassion Scale | Narcissism [Narcissistic Personality Inventory]/  *r* = 0.003 | Well-being [Well-being Index]/  *r* = 0.38** | *r* = 0.17** | 420 students | Cross-sectional | AMOS/SEM | ***Well-being [outcome]***  Narcissism: indirect effect = -0.04 [-0.07, -0.01]/*PM* = 0.19 |  |
| Yang et al., 2016 | Self-Compassion Scale | Hope [State Hope Scale]/  *r* = 0.45*** | Life satisfaction [Satisfaction with Life Scale]/  *r* = 0.36*** | *r* = 0.64*** | 320 adults | Cross-sectional | Mplus/SEM | ***Life satisfaction [outcome]***  Hope: indirect effect = 0.33 [0.23, 0.43]/*PM* = 0.77 |  |
| Tran et al., 2024 | Self-Compassion Scale | Hope [The State Hope Scale] r = 0.58** | Life Satisfaction [The Satisfaction With Life Scale] r = 0.47**  Psychological Well-Being [The World Health Organization 5-item Well-Being Index] r = 0.52** | Life Satisfaction: r = 0.70**  Psychological Well-Being: r = 0.69** | 484 undergraduate students | Cross-sectional | SEM | ***Life Satisfaction [outcome]***  Indirect effect = 0.44 [0.36, 0.53]  ***Psychological Well-Being [outcome]***  Indirect effect = 0.52 [0.43, 0.61] |  |
| Sperandio et al., 2022 | Self-Compassion Scale-Short Form | Hope [Adult Hope Scale]/  *r* = 0.44** | Posttraumatic Growth [Posttraumatic Growth Inventory]/  *r* = 0.27** | *r* = 0.62** | 292 individuals who experienced the drug related death of a loved one | Cross-sectional | AMOS/SEM | ***[self-compassion subscales as independent variables]***  Positive self-compassion - Hope - PTG: indirect effect = 0.37, p < 0.01/Not applicable; Negative self-compassion - Hope - PTG: indirect effect is not significant [parameters undisclosed] |  |
| Liu et al., 2024 | Self-Compassion Scale-Short Form | Hope [The Hope Scale] *r* = 0.50** | Flourishing [Flourishing Scale] *r* = 0.53** | *r* = 0.41** | 842 adults | Cross-sectional | Mediation analysis | ***Flourishing [outcome]***  Indirect effect = 0.20 [0.15, 0.25]/*PM* = 0.38 |  |
| Pyszkowska & Rönnlund, 2021 | Self-Compassion Scale | Balanced time perspective [Zimbardo Time Perspective Inventory]/  *r* = -0.44** | Well-being [Satisfaction with Life Scale]/  *r* = 0.45** | *r* = -0.51*** | 431 individuals | Cross-sectional | AMOS/SEM | ***Well-being [outcome]***  Balanced time perspective : indirect effect = 0.22, p < 0.001/*PM* = 0.40 |  |
| Wu et al., 2022 | Self-Compassion Scale-Short Form | Meaning of Life [Meaning in Life Questionnaire]  *r* = 0.41*** | Happiness [The subjective authentic-durable happiness scale]  *r* = 0.45*** | *r* = 0.39*** | 1165 college students | Cross-sectional | Mediation analysis | ***Happiness [outcome]***  Indirect effect = 0.10 [0.04, 0.10]/*PM* = 0.22 |  |
| Zeng et al., 2023 | Self-Compassion Scale-Short Form | Prosocial behaviour  [The Internet Altruistic Behaviour Scale of Undergraduates] *r* = 0.17** | Subjective well‐being [The Index of Well‐being] *r* = 0.44** | *r* = 0.20** | 1488 Participants | Cross-sectional | Mediation analysis | ***Well-being [outcome]***  Indirect effect = 0.02 [0.01, 0.03]/*PM* = 0.05 |  |
| Min et al., 2022 | Self-Compassion Scale | Help-seeking behaviour [the Actual Help-Seeking Questionnaire] *r* = 0.14** | Flourishing [Flourishing Scale] *r* = 0.51 | *r* = -0.07 | 605 students | Cross-sectional | SEM | ***Flourishing [outcome]***  Indirect effect = 0.02/*PM* = 0.03 |  |
| Li & Wang, 2024 | Self-Compassion Scale | Perceived control [The Psychological Security Questionnaire subscale] *r* = 0.46** | General well-being [General well-being Scale] *r* = 0.39** | *r* = 0.31** | 355 participants | Cross-sectional | Mediation analysis | ***Well-being [outcome]***  Indirect effect = 0.19 [0.07, 0.31]/*PM* = 0.23 |  |
| Xie, 2023 | Self-Compassion Scale | Non-attachment [The Nonattachment Scale] *r* = 0.68** | Peace [The 7-item Peace of Mind Scale] *r* = 0.71** | *r* = 0.72** | 364 participants | Cross-sectional | Mediation analysis | ***Peace [outcome]***  Indirect effect = 0.30 [0.23, 0.36]/*PM* = 0.32 |  |

Note: **p* < 0.05, ***p* < 0.01, ****p*<0.001.

***Appendix B: Quality assessment results of included studies***

| **Author and year** | Items | | | | | | | | | | | Total Score |
| --- | --- | --- | --- | --- | --- | --- | --- | --- | --- | --- | --- | --- |
|  | 1 | 2 | 3 | 4 | 5 | 6 | 7 | 8 | 9 | 10 | 11 |  |
| Ozonder Unal & Ordu, 2023 | 0 | 0 | 0 | 0 | 0 | 0 | 0 | 1 | 0 | 0 | 0 | 1 |
| Özönder Ünal et al., 2023 | 0 | 0 | 0 | 0 | 0 | 1 | 0 | 1 | 0 | 0 | 0 | 2 |
| Mosewich et al., 2019 | 0 | 0 | 0 | 0 | 1 | 1 | 0 | 1 | 1 | 0 | 0 | 4 |
| Carvalho et al., 2018 | 0 | 0 | 0 | 1 | 1 | 1 | 0 | 1 | 0 | 0 | 0 | 4 |
| Makadi & Koszycki, 2020 | 0 | 0 | 0 | 1 | 1 | 1 | 0 | 1 | 0 | 0 | 0 | 4 |
| Phillips, 2018 | 0 | 0 | 0 | 1 | 1 | 1 | 0 | 1 | 0 | 0 | 0 | 4 |
| Morgenroth et al., 2022 | 1 | 0 | 0 | 1 | 1 | 0 | 0 | 1 | 0 | 0 | 0 | 4 |
| Hatun & Kurtça, 2023 | 0 | 0 | 0 | 1 | 1 | 1 | 0 | 1 | 0 | 0 | 0 | 4 |
| Roxas et al., 2019 | 0 | 0 | 0 | 1 | 1 | 1 | 0 | 1 | 0 | 0 | 0 | 4 |
| Ericson et al., 2024 | 0 | 0 | 0 | 1 | 1 | 1 | 0 | 1 | 0 | 0 | 0 | 4 |
| Eghbali et al., 2022 | 0 | 0 | 0 | 1 | 1 | 1 | 0 | 1 | 0 | 0 | 0 | 4 |
| Zhu et al., 2022 | 0 | 0 | 0 | 1 | 1 | 1 | 0 | 1 | 0 | 0 | 0 | 4 |
| Salehi et al., 2023 | 0 | 0 | 0 | 1 | 1 | 1 | 0 | 1 | 0 | 0 | 0 | 4 |
| Bogerd et al., 2023 | 0 | 0 | 0 | 1 | 1 | 1 | 0 | 1 | 0 | 0 | 0 | 4 |
| Zerach, 2025 | 0 | 0 | 0 | 1 | 1 | 1 | 0 | 1 | 0 | 0 | 0 | 4 |
| Karataş & Tüccar, 2025 | 0 | 0 | 0 | 1 | 1 | 1 | 0 | 1 | 0 | 0 | 0 | 4 |
| Julian et al., 2025 | 0 | 0 | 0 | 1 | 1 | 1 | 0 | 1 | 0 | 0 | 0 | 4 |
| Maddock, 2024a, 2025 | 0 | 0 | 0 | 1 | 1 | 1 | 0 | 1 | 0 | 0 | 0 | 4 |
| Beato et al., 2021 | 1 | 0 | 0 | 1 | 1 | 1 | 0 | 1 | 0 | 0 | 0 | 5 |
| Eichholz et al., 2020 | 1 | 0 | 0 | 1 | 1 | 1 | 0 | 1 | 0 | 0 | 0 | 5 |
| Chase et al., 2019 | 0 | 0 | 0 | 1 | 1 | 1 | 1 | 1 | 0 | 0 | 0 | 5 |
| Murfield et al., 2020 | 0 | 0 | 1 | 1 | 1 | 1 | 0 | 1 | 0 | 0 | 0 | 5 |
| Finlay-Jones et al., 2015 | 1 | 0 | 0 | 1 | 1 | 1 | 0 | 1 | 0 | 0 | 0 | 5 |
| Carona et al., 2022 | 1 | 0 | 0 | 1 | 1 | 1 | 0 | 1 | 0 | 0 | 0 | 5 |
| S. L. Brown et al., 2020 | 1 | 0 | 0 | 1 | 1 | 1 | 0 | 1 | 0 | 0 | 0 | 5 |
| Bakker et al., 2019 | 1 | 0 | 0 | 1 | 1 | 1 | 0 | 1 | 0 | 0 | 0 | 5 |
| Raes, 2010 | 1 | 0 | 0 | 1 | 1 | 1 | 0 | 1 | 0 | 0 | 0 | 5 |
| Jansen, 2021 | 0 | 0 | 0 | 1 | 1 | 1 | 1 | 1 | 0 | 0 | 0 | 5 |
| Jansen, Siebertz, et al., 2021 | 0 | 0 | 0 | 1 | 1 | 1 | 1 | 1 | 0 | 0 | 0 | 5 |
| Rakhimov et al., 2023 | 1 | 0 | 0 | 1 | 1 | 1 | 0 | 1 | 0 | 0 | 0 | 5 |
| Lenferink et al., 2017 | 1 | 0 | 0 | 1 | 1 | 1 | 0 | 1 | 0 | 0 | 0 | 5 |
| Hodgetts et al., 2021 | 1 | 0 | 0 | 1 | 1 | 1 | 0 | 1 | 0 | 0 | 0 | 5 |
| Fresnics & Borders, 2017 | 1 | 0 | 0 | 1 | 1 | 1 | 0 | 1 | 0 | 0 | 0 | 5 |
| Casali et al., 2022 | 1 | 0 | 0 | 1 | 1 | 1 | 0 | 1 | 0 | 0 | 0 | 5 |
| Krieger et al., 2013 | 1 | 0 | 0 | 1 | 1 | 1 | 0 | 1 | 0 | 0 | 0 | 5 |
| Bates et al., 2021 | 1 | 0 | 0 | 1 | 1 | 1 | 0 | 1 | 0 | 0 | 0 | 5 |
| Pérez-Aranda et al., 2021 | 1 | 0 | 0 | 1 | 1 | 1 | 0 | 1 | 0 | 0 | 0 | 5 |
| Zhao et al., 2022 | 1 | 0 | 0 | 1 | 1 | 1 | 0 | 1 | 0 | 0 | 0 | 5 |
| Zhou et al., 2013 | 1 | 0 | 0 | 1 | 1 | 1 | 0 | 1 | 0 | 0 | 0 | 5 |
| Arimitsu & Hofmann, 2015 | 1 | 0 | 0 | 1 | 1 | 1 | 0 | 1 | 0 | 0 | 0 | 5 |
| Angus & Phillips, 2021 | 1 | 0 | 0 | 1 | 1 | 1 | 0 | 1 | 0 | 0 | 0 | 5 |
| Ghorbani et al., 2012 | 1 | 0 | 0 | 1 | 1 | 1 | 0 | 1 | 0 | 0 | 0 | 5 |
| Brown-Beresford & McLaren, 2022 | 1 | 0 | 0 | 1 | 1 | 1 | 0 | 1 | 0 | 0 | 0 | 5 |
| Zhu et al., 2020 | 1 | 0 | 0 | 1 | 1 | 1 | 0 | 1 | 0 | 0 | 0 | 5 |
| Gerber et al., 2021 | 1 | 0 | 0 | 1 | 1 | 0 | 1 | 1 | 0 | 0 | 0 | 5 |
| Stephenson et al., 2018 | 1 | 0 | 0 | 1 | 1 | 1 | 0 | 1 | 0 | 0 | 0 | 5 |
| Deniz, 2021 | 1 | 0 | 0 | 1 | 1 | 1 | 0 | 1 | 0 | 0 | 0 | 5 |
| Voon et al., 2022 | 1 | 0 | 0 | 1 | 1 | 1 | 0 | 1 | 0 | 0 | 0 | 5 |
| Wong & Yeung, 2017 | 1 | 0 | 0 | 1 | 1 | 1 | 0 | 1 | 0 | 0 | 0 | 5 |
| Munroe et al., 2022 | 1 | 0 | 0 | 1 | 1 | 1 | 0 | 1 | 0 | 0 | 0 | 5 |
| Pyszkowska & Rönnlund, 2021 | 1 | 0 | 0 | 1 | 1 | 1 | 0 | 1 | 0 | 0 | 0 | 5 |
| L. Brown et al., 2016 | 1 | 0 | 0 | 1 | 1 | 1 | 0 | 1 | 0 | 0 | 0 | 5 |
| Quang et al., 2022 | 1 | 0 | 0 | 1 | 1 | 1 | 0 | 1 | 0 | 0 | 0 | 5 |
| Yang et al., 2016 | 1 | 0 | 0 | 1 | 1 | 1 | 0 | 1 | 0 | 0 | 0 | 5 |
| Sperandio et al., 2022 | 1 | 0 | 0 | 1 | 1 | 1 | 0 | 1 | 0 | 0 | 0 | 5 |
| Wu et al., 2022 | 1 | 0 | 0 | 1 | 1 | 1 | 0 | 1 | 0 | 0 | 0 | 5 |
| Zeng et al., 2023 | 1 | 0 | 0 | 1 | 1 | 1 | 0 | 1 | 0 | 0 | 0 | 5 |
| Tran et al., 2024 | 1 | 0 | 0 | 1 | 1 | 1 | 0 | 1 | 0 | 0 | 0 | 5 |
| Min et al., 2022 | 1 | 0 | 0 | 1 | 1 | 1 | 0 | 1 | 0 | 0 | 0 | 5 |
| Li & Wang, 2024 | 0 | 0 | 0 | 1 | 1 | 1 | 1 | 1 | 0 | 0 | 0 | 5 |
| Xie, 2023 | 1 | 0 | 0 | 1 | 1 | 1 | 0 | 1 | 0 | 0 | 0 | 5 |
| Xie et al., 2023 | 1 | 0 | 0 | 1 | 1 | 1 | 0 | 1 | 0 | 0 | 0 | 5 |
| Cai et al., 2023 | 1 | 0 | 0 | 1 | 1 | 1 | 0 | 1 | 0 | 0 | 0 | 5 |
| Ueno & Amemiya, 2024 | 0 | 0 | 0 | 0 | 0 | 0 | 1 | 1 | 1 | 1 | 1 | 5 |
| Skinner & Kuijer, 2024 | 1 | 0 | 0 | 1 | 1 | 1 | 0 | 1 | 0 | 0 | 0 | 5 |
| Pyszkowska et al., 2024 | 1 | 0 | 0 | 1 | 1 | 1 | 0 | 1 | 0 | 0 | 0 | 5 |
| Cutajar & Bates, 2025 | 0 | 0 | 0 | 1 | 1 | 1 | 1 | 1 | 0 | 0 | 0 | 5 |
| Hou et al., 2025 | 1 | 0 | 0 | 1 | 1 | 1 | 0 | 1 | 0 | 0 | 0 | 5 |
| Kaçar-Başaran, 2025; Kaçar-Başaran & Gökdağ, 2025 | 1 | 0 | 0 | 1 | 1 | 1 | 0 | 1 | 0 | 0 | 0 | 5 |
| Liu et al., 2024 | 1 | 0 | 0 | 1 | 1 | 1 | 0 | 1 | 0 | 0 | 0 | 5 |
| Kaya et al., 2024 | 0 | 0 | 0 | 1 | 1 | 1 | 1 | 1 | 0 | 0 | 0 | 5 |
| Wang et al., 2024 | 1 | 0 | 0 | 1 | 1 | 1 | 0 | 1 | 0 | 0 | 0 | 5 |
| Ristvej et al., 2024 | 1 | 0 | 0 | 1 | 1 | 1 | 0 | 1 | 0 | 0 | 0 | 5 |
| Blankenship & Hogge, 2024 | 1 | 0 | 0 | 1 | 1 | 1 | 0 | 1 | 0 | 0 | 0 | 5 |
| Akdeniz & Birekul, 2024 | 0 | 0 | 0 | 1 | 1 | 1 | 1 | 1 | 0 | 0 | 0 | 5 |
| Vidal et al., 2024 | 1 | 0 | 0 | 1 | 1 | 1 | 0 | 1 | 0 | 0 | 0 | 5 |
| Chong & Chan, 2023 | 1 | 0 | 0 | 1 | 1 | 1 | 0 | 1 | 0 | 0 | 0 | 5 |
| Rehman et al., 2024 | 0 | 0 | 0 | 1 | 1 | 1 | 1 | 1 | 0 | 0 | 0 | 5 |
| Ewert et al., 2018 | 1 | 0 | 0 | 1 | 1 | 1 | 0 | 1 | 1 | 0 | 0 | 6 |
| Johnson & O’Brien, 2013 | 1 | 0 | 0 | 1 | 1 | 1 | 1 | 1 | 0 | 0 | 0 | 6 |
| Hamrick & Owens, 2019 | 1 | 0 | 0 | 1 | 1 | 1 | 1 | 1 | 0 | 0 | 0 | 6 |
| Adie et al., 2021 | 1 | 0 | 0 | 1 | 1 | 1 | 1 | 1 | 0 | 0 | 0 | 6 |
| Gouveia et al., 2016 | 1 | 0 | 0 | 1 | 1 | 1 | 1 | 1 | 0 | 0 | 0 | 6 |
| Norman et al., 2020 | 1 | 0 | 0 | 1 | 1 | 1 | 1 | 1 | 0 | 0 | 0 | 6 |
| Ying, 2009 | 1 | 0 | 0 | 1 | 1 | 1 | 1 | 1 | 0 | 0 | 0 | 6 |
| Eccles et al., 2023 | 1 | 0 | 0 | 1 | 1 | 1 | 1 | 1 | 0 | 0 | 0 | 6 |
| Gerber & Anaki, 2021 | 1 | 0 | 0 | 1 | 1 | 1 | 1 | 1 | 0 | 0 | 0 | 6 |
| Shin, 2019 | 1 | 0 | 0 | 1 | 1 | 1 | 1 | 1 | 0 | 0 | 0 | 6 |
| Li et al., 2021 | 1 | 0 | 0 | 1 | 1 | 1 | 1 | 1 | 0 | 0 | 0 | 6 |
| Nguyen & Le, 2021 | 1 | 0 | 1 | 1 | 1 | 1 | 0 | 1 | 0 | 0 | 0 | 6 |
| McBride et al., 2022 | 1 | 0 | 0 | 1 | 1 | 1 | 1 | 1 | 0 | 0 | 0 | 6 |
| Zipagan & Galvez Tan, 2023 | 1 | 0 | 0 | 1 | 1 | 1 | 1 | 1 | 0 | 0 | 0 | 6 |
| Coutts et al., 2023 | 1 | 0 | 0 | 1 | 1 | 1 | 1 | 1 | 0 | 0 | 0 | 6 |
| Maddock et al., 2020 | 1 | 0 | 0 | 1 | 1 | 1 | 1 | 1 | 1 | 0 | 0 | 7 |
| Hsieh et al., 2021 | 1 | 0 | 0 | 1 | 1 | 1 | 0 | 1 | 1 | 0 | 1 | 7 |
| Xu et al., 2024 | 0 | 1 | 0 | 1 | 1 | 1 | 0 | 1 | 1 | 1 | 0 | 7 |
| Maddock, 2024b | 0 | 1 | 0 | 1 | 1 | 1 | 0 | 1 | 1 | 1 | 0 | 7 |
| Allen et al., 2024 | 1 | 0 | 0 | 1 | 1 | 1 | 0 | 1 | 1 | 0 | 1 | 7 |
| Townshend & Caltabiano, 2019 | 0 | 1 | 0 | 1 | 1 | 1 | 1 | 1 | 1 | 0 | 0 | 7 |
| Jansen, Hoja, et al., 2021 | 1 | 1 | 0 | 1 | 1 | 1 | 1 | 1 | 0 | 0 | 0 | 7 |
| Lefebvre et al., 2021 | 1 | 0 | 0 | 1 | 1 | 1 | 1 | 1 | 1 | 0 | 1 | 8 |
| Diedrich et al., 2017 | 1 | 0 | 0 | 1 | 1 | 1 | 1 | 1 | 1 | 0 | 1 | 8 |
| Ewert et al., 2022 | 1 | 0 | 0 | 1 | 1 | 1 | 0 | 1 | 1 | 1 | 1 | 8 |
| Zhang et al., 2025 | 1 | 0 | 0 | 1 | 1 | 1 | 0 | 1 | 1 | 1 | 1 | 8 |
| Shi et al., 2025 | 1 | 0 | 0 | 1 | 1 | 1 | 0 | 1 | 1 | 1 | 1 | 8 |
| Peng & Ishak, 2024 | 1 | 0 | 0 | 1 | 1 | 1 | 0 | 1 | 1 | 1 | 1 | 8 |
| Cabaços et al., 2023 | 1 | 0 | 0 | 1 | 1 | 1 | 0 | 1 | 1 | 1 | 1 | 8 |
| Kreemers et al., 2020 | 1 | 1 | 1 | 1 | 1 | 1 | 0 | 1 | 1 | 0 | 0 | 8 |
| Wadsworth et al., 2018 | 1 | 1 | 0 | 1 | 1 | 1 | 0 | 1 | 1 | 1 | 0 | 8 |
| Ewert et al., 2024 (Study 1) | 1 | 0 | 0 | 1 | 1 | 1 | 1 | 1 | 1 | 1 | 1 | 9 |
| Asselmann et al., 2024 | 1 | 0 | 0 | 1 | 1 | 1 | 1 | 1 | 1 | 1 | 1 | 9 |
| Yela et al., 2022 | 1 | 1 | 0 | 1 | 1 | 1 | 1 | 1 | 1 | 1 | 0 | 9 |
| Ewert et al., 2024 (Study 2) | 1 | 0 | 1 | 1 | 1 | 1 | 1 | 1 | 1 | 1 | 1 | 10 |

1. Did the study include ***a theoretical framework***?
2. Were the study ***methods/procedures designed to influence mediating variables***?
3. Did the authors report conducting ***pilot studies*** to test mediation?
4. Were the measures of ***self-compassion reliable***? (Cronbach's alpha > 0.60)
5. Were the measures of ***psychological outcome reliable***? (Cronbach's alpha > 0.60)
6. Were the ***mediator measures reliable***? (Cronbach's alpha > 0.60)
7. Did the study report a ***power calculation***, and was the study powered to detect mediation?
8. Were ***statistically appropriate/acceptable methods*** of data analyses used?
9. Was the design ***a longitudinal/interventional/experimental design***?
10. Was ***baseline psychological outcome*** considered in analyses?
11. Did the study ascertain whether ***changes in the mediator precede changes in psychological outcome***?

**Reference**

Adie, T., Steindl, S. R., Kirby, J. N., Kane, R. T., & Mazzucchelli, T. G. (2021). The Relationship Between Self-Compassion and Depressive Symptoms: Avoidance and Activation as Mediators. *Mindfulness*, *12*(7), 1748–1756. https://doi.org/10.1007/s12671-021-01637-1

Akdeniz, S., & Birekul, M. (2024). The Mediating Role of Difficulties in Emotion Regulation and Social Anxiety in the Relationship between Self-Compassion and Internet Addiction. *Studia Psychologica*, *66*(4), 253–266. https://doi.org/10.31577/sp.2024.04.904

Allen, A. B., Littleton, H., Bistricky, S., Benson, K., Cox, T., & Benight, C. C. (2024). Self-compassion reduces posttraumatic stress symptom severity in hurricane survivors via perceived social support. *Psychological Trauma: Theory, Research, Practice, and Policy*, *16*(Suppl 3), S582–S589. https://doi.org/10.1037/tra0001630

Angus, B. M., & Phillips, W. J. (2021). Self-referent upward counterfactual thinking mediates the relationship between self-compassion and depression. *Australian Psychologist*, *56*(1), 61–69. https://doi.org/10.1080/00050067.2021.1890980

Arimitsu, K., & Hofmann, S. G. (2015). Cognitions as mediators in the relationship between self-compassion and affect. *Personality and Individual Differences*, *74*, 41–48. https://doi.org/10.1016/j.paid.2014.10.008

Asselmann, E., Bendau, A., Hoffmann, C., & Ewert, C. (2024). Self-compassion Predicts Higher Affective Well-being and Lower Stress Symptoms Through Less Dysfunctional Coping: A Three-wave Longitudinal Study During the COVID-19 Pandemic. *Journal of Happiness Studies*, *25*(5), 55. https://doi.org/10.1007/s10902-024-00755-6

Bakker, A. M., Cox, D. W., Hubley, A. M., & Owens, R. L. (2019). Emotion Regulation as a Mediator of Self-Compassion and Depressive Symptoms in Recurrent Depression. *Mindfulness*, *10*(6), 1169–1180. https://doi.org/10.1007/s12671-018-1072-3

Bates, G. W., Elphinstone, B., & Whitehead, R. (2021). Self‐compassion and emotional regulation as predictors of social anxiety. *Psychology and Psychotherapy: Theory, Research and Practice*, *94*(3), 426–442. https://doi.org/10.1111/papt.12318

Beato, A. F., da Costa, L. P., & Nogueira, R. (2021). “Everything Is Gonna Be Alright with Me”: The Role of Self-Compassion, Affect, and Coping in Negative Emotional Symptoms during Coronavirus Quarantine. *International Journal of Environmental Research and Public Health*, *18*(4), 2017. https://doi.org/10.3390/ijerph18042017

Blankenship, P., & Hogge, I. (2024). Self-Compassion and Psychological Well-Being of Childhood Sexual Abuse Survivors: Emotional Dysregulation and Trauma-Related Shame as Mediators. *Journal of Interpersonal Violence*. https://doi.org/10.1177/08862605241268781

Bogerd, R., Debets, M. P. M., Keuken, D. G., Hassink, R. J., Henriques, J. P. S., & Lombarts, K. M. J. M. H. (2023). The relationship between physicians’ self-kindness and professional fulfillment and the mediating role of personal resilience and work-home interference: A cross-sectional study. *PLOS ONE*, *18*(4), e0284507. https://doi.org/10.1371/journal.pone.0284507

Brown, L., Bryant, C., Brown, V., Bei, B., & Judd, F. (2016). Self-compassion, attitudes to ageing and indicators of health and well-being among midlife women. *Aging & Mental Health*, *20*(10), 1035–1043. https://doi.org/10.1080/13607863.2015.1060946

Brown, S. L., Hughes, M., Campbell, S., & Cherry, M. G. (2020). Could worry and rumination mediate relationships between self‐compassion and psychological distress in breast cancer survivors? *Clinical Psychology & Psychotherapy*, *27*(1), 1–10. https://doi.org/10.1002/cpp.2399

Brown-Beresford, E., & McLaren, S. (2022). The Relationship between Self-Compassion, Internalized Heterosexism, and Depressive Symptoms among Bisexual and Lesbian Women. *Journal of Bisexuality*, *22*(1), 90–115. https://doi.org/10.1080/15299716.2021.2004483

Cabaços, C., Macedo, A., Carneiro, M., Brito, M. J., Amaral, A. P., Araújo, A., Correia, D. T., Novais, F., Vitória, P., & Pereira, A. T. (2023). The mediating role of self-compassion and repetitive negative thinking in the relationship between perfectionism and burnout in health-field students: A prospective study. *Personality and Individual Differences*, *213*, 112314. https://doi.org/10.1016/j.paid.2023.112314

Cai, R. Y., Love, A., Robinson, A., & Gibbs, V. (2023). The Inter-Relationship of Emotion Regulation, Self-Compassion, and Mental Health in Autistic Adults. *Autism in Adulthood*, *5*(3), 335–342. https://doi.org/10.1089/aut.2022.0068

Carona, C., Xavier, S., Canavarro, M. C., & Fonseca, A. (2022). Self‐compassion and complete perinatal mental health in women at high risk for postpartum depression: The mediating role of emotion regulation difficulties. *Psychology and Psychotherapy: Theory, Research and Practice*, *95*(2), 561–574. https://doi.org/10.1111/papt.12388

Carvalho, S. A., Gillanders, D., Palmeira, L., Pinto‐Gouveia, J., & Castilho, P. (2018). Mindfulness, selfcompassion, and depressive symptoms in chronic pain: The role of pain acceptance. *Journal of Clinical Psychology*, *74*(12), 2094–2106. https://doi.org/10.1002/jclp.22689

Casali, N., Ghisi, M., Jansen, P., Feraco, T., & Meneghetti, C. (2022). What Can Affect Competition Anxiety in Athletes? The Role of Self-Compassion and Repetitive Negative Thinking. *Psychological Reports*, *125*(4), 2009–2028. https://doi.org/10.1177/00332941211017258

Chase, T. E., Chasson, G. S., Hamilton, C. E., Wetterneck, C. T., Smith, A. H., & Hart, J. M. (2019). The Mediating Role of Emotion Regulation Difficulties in the Relationship Between Self-Compassion and OCD Severity in a Non-Referred Sample. *Journal of Cognitive Psychotherapy*, *33*(2), 157–168. https://doi.org/10.1891/0889-8391.33.2.157

Chong, E. S. K., & Chan, R. C. H. (2023). The Role of Self-Compassion in Minority Stress Processes and Life Satisfaction among Sexual Minorities in Hong Kong. *Mindfulness*, *14*(4), 784–796. https://doi.org/10.1007/s12671-023-02106-7

Coutts, J. J., Al-Kire, R. L., & Weidler, D. J. (2023). I can see (myself) clearly now: Exploring the mediating role of self-concept clarity in the association between self-compassion and indicators of well-being. *PLOS ONE*, *18*(6), e0286992. https://doi.org/10.1371/journal.pone.0286992

Cutajar, K., & Bates, G. W. (2025). Australian Women in the Perinatal Period During COVID-19: The Influence of Self-Compassion and Emotional Regulation on Anxiety, Depression, and Social Anxiety. *Healthcare*, *13*(2), 120. https://doi.org/10.3390/healthcare13020120

Deniz, M. E. (2021). Self-compassion, intolerance of uncertainty, fear of COVID-19, and well-being: A serial mediation investigation. *Personality and Individual Differences*, *177*, 110824. https://doi.org/10.1016/j.paid.2021.110824

Diedrich, A., Burger, J., Kirchner, M., & Berking, M. (2017). Adaptive emotion regulation mediates the relationship between self‐compassion and depression in individuals with unipolar depression. *Psychology and Psychotherapy: Theory, Research and Practice*, *90*(3), 247–263. https://doi.org/10.1111/papt.12107

Eccles, F. J. R., Sowter, N., Spokes, T., Zarotti, N., & Simpson, J. (2023). Stigma, self-compassion, and psychological distress among people with Parkinson’s. *Disability and Rehabilitation*, *45*(3), 425–433. https://doi.org/10.1080/09638288.2022.2037743

Eghbali, B., Saadat, S., Hasanzadeh, K., Pourramzani, A., Khatami, S., Saberi, A., & Jafroudi, M. (2022). Relationship between self-compassion and psychological well-being with the mediating role of resilience in people with multiple sclerosis. *Postępy Psychiatrii i Neurologii*, *31*(2), 43–51. https://doi.org/10.5114/ppn.2022.117999

Eichholz, A., Schwartz, C., Meule, A., Heese, J., Neumüller, J., & Voderholzer, U. (2020). Self‐compassion and emotion regulation difficulties in obsessive–compulsive disorder. *Clinical Psychology & Psychotherapy*, *27*(5), 630–639. https://doi.org/10.1002/cpp.2451

Ericson, S. M., Gallagher, J. P., Federico, A. J., Fleming, J. J., Froggatt, D., Eleid, A., Finn, B. M., Johnston, K., & Cai, R. Y. (2024). Does emotion regulation mediate the relationship between self-compassion and subjective well-being? A cross-sectional study of adults living in the United States. *Journal of Health Psychology*, *29*(8), 863–876. https://doi.org/10.1177/13591053231209668

Ewert, C., Buechner, A., & Schröder-Abé, M. (2024). Stress Perception and Coping as Mediators of the Link Between Self-Compassion and Affective Well-being? Evidence From Two Longitudinal Studies. *Mindfulness*, *15*(2), 372–388. https://doi.org/10.1007/s12671-023-02295-1

Ewert, C., Gaube, B., & Geisler, F. C. M. (2018). Dispositional self-compassion impacts immediate and delayed reactions to social evaluation. *Personality and Individual Differences*, *125*, 91–96. https://doi.org/10.1016/j.paid.2017.12.037

Ewert, C., Hoffmann, C. F. A., & Schröder-Abé, M. (2022). Stress Processing Mediates the Link Between Momentary Self-compassion and Affective Well-being. *Mindfulness*, *13*(9), 2269–2281. https://doi.org/10.1007/s12671-022-01954-z

Finlay-Jones, A., Rees, C. S., & Kane, R. T. (2015). Self-Compassion, Emotion Regulation and Stress among Australian Psychologists: Testing an Emotion Regulation Model of Self-Compassion Using Structural Equation Modeling. *PLOS ONE*, *10*(7), e0133481. https://doi.org/10.1371/journal.pone.0133481

Fresnics, A., & Borders, A. (2017). Angry Rumination Mediates the Unique Associations Between Self-Compassion and Anger and Aggression. *Mindfulness*, *8*(3), 554–564. https://doi.org/10.1007/s12671-016-0629-2

Gerber, Z., & Anaki, D. (2021). The Role of Self-compassion, Concern for Others, and Basic Psychological Needs in the Reduction of Caregiving Burnout. *Mindfulness*, *12*(3), 741–750. https://doi.org/10.1007/s12671-020-01540-1

Gerber, Z., Davidovics, Z., & Anaki, D. (2021). The Relationship Between Self-Compassion, Concern for Others, and Parental Burnout in Child’s Chronic Care Management. *Mindfulness*, *12*(12), 2920–2928. https://doi.org/10.1007/s12671-021-01752-z

Ghorbani, N., Watson, P. J., Chen, Z., & Norballa, F. (2012). Self-Compassion in Iranian Muslims: Relationships With Integrative Self-Knowledge, Mental Health, and Religious Orientation. *International Journal for the Psychology of Religion*, *22*(2), 106–118. https://doi.org/10.1080/10508619.2011.638601

Gouveia, M. J., Carona, C., Canavarro, M. C., & Moreira, H. (2016). Self-Compassion and Dispositional Mindfulness Are Associated with Parenting Styles and Parenting Stress: the Mediating Role of Mindful Parenting. *Mindfulness*, *7*(3), 700–712. https://doi.org/10.1007/s12671-016-0507-y

Hamrick, L. A., & Owens, G. P. (2019). Exploring the mediating role of self‐blame and coping in the relationships between self‐compassion and distress in females following the sexual assault. *Journal of Clinical Psychology*, *75*(4), 766–779. https://doi.org/10.1002/jclp.22730

Hatun, O., & Kurtça, T. T. (2023). Self-compassion, Resilience, Fear of COVID-19, Psychological Distress, and Psychological Well-being among Turkish Adults. *Current Psychology*, *42*(23), 20052–20062. https://doi.org/10.1007/s12144-022-02824-6

Hodgetts, J., McLaren, S., Bice, B., & Trezise, A. (2021). The relationships between self-compassion, rumination, and depressive symptoms among older adults: the moderating role of gender. *Aging & Mental Health*, *25*(12), 2337–2346. https://doi.org/10.1080/13607863.2020.1824207

Hou, J., Qu, D., Bu, H., Chen, B., Liu, G., & Yu, N. X. (2025). The association between self-compassion and psychological distress among Chinese college students: serial mediation via emotions and resilience resources. *Current Psychology*, *44*(2), 1324–1332. https://doi.org/10.1007/s12144-024-07227-3

Hsieh, C.-C., Lin, Z.-Z., Ho, C.-C., Yu, C.-J., Chen, H.-J., Chen, Y.-W., & Hsiao, F.-H. (2021). The Short- and Long-term Causal Relationships Between Self-compassion, Trait Mindfulness, Caregiver Stress, and Depressive Symptoms in Family Caregivers of Patients with Lung Cancer. *Mindfulness*, *12*(7), 1812–1821. https://doi.org/10.1007/s12671-021-01642-4

Jansen, P. (2021). Self-compassion and repetitive thinking in relation to depressive mood and fear of the future. *German Journal of Exercise and Sport Research*, *51*(2), 232–236. https://doi.org/10.1007/s12662-021-00712-y

Jansen, P., Hoja, S., & Meneghetti, C. (2021). Does repetitive thinking mediate the relationship between self-compassion and competition anxiety in athletes? *Cogent Psychology*, *8*(1). https://doi.org/10.1080/23311908.2021.1909243

Jansen, P., Siebertz, M., Hofmann, P., Zayed, K., Zayed, D., Abdelfattah, F., Fernández-Méndez, L. M., & Meneghetti, C. (2021). Does self-compassion relate to the fear of the future during the 2020 coronavirus pandemic? A cross-cultural study. *Cogent Psychology*, *8*(1). https://doi.org/10.1080/23311908.2021.1976438

Johnson, E. A., & O’Brien, K. A. (2013). Self-compassion soothes the savage ego-threat system: Effects on negative affect, shame, rumination, and depressive symptoms. *Journal of Social and Clinical Psychology*, *32*(9), 939–963.

Julian, K., Allbaugh, L. J., Selvey-Bouyack, A. M., & Lutz-Zois, C. (2025). Self-Compassion, Mindfulness, and Emotion Regulation: Understanding the Relationship Between Childhood Maltreatment and Wellbeing. *Journal of Aggression, Maltreatment & Trauma*, *34*(2), 222–241. https://doi.org/10.1080/10926771.2025.2452921

Kaçar-Başaran, S. (2025). From Hero to Zero: A Serial Mediation Model Between Narcissistic Perfectionism and Psychological Distress. *Journal of Rational-Emotive & Cognitive-Behavior Therapy*, *43*(1), 4. https://doi.org/10.1007/s10942-024-00572-z

Kaçar-Başaran, S., & Gökdağ, C. (2025). From self-compassion to obsessive-compulsive symptoms: the mediator role of intolerance of uncertainty. *Current Psychology*, *44*(4), 2375–2384. https://doi.org/10.1007/s12144-025-07324-x

Karataş, Z., & Tüccar, E. (2025). The Mediating Effect of Rumination in the Relationship between Secondary Traumatic Stress and Self-Compassion in Psychosocial Support Providers. *Journal of Social Service Research*, *51*(3), 801–815. https://doi.org/10.1080/01488376.2024.2407606

Kaya, Y., Osmanoğlu, N., Satıcı, S. A., & Deniz, M. E. (2024). Self-compassion and death distress among individuals affected by the February 6, 2023, Türkiye Earthquake: The mediating role of doomscrolling and resilience. *Journal of Health Psychology*. https://doi.org/10.1177/13591053241282129

Kreemers, L. M., van Hooft, E. A. J., van Vianen, A. E. M., & Sisouw de Zilwa, S. C. M. (2020). Testing a Self-Compassion Intervention Among Job Seekers: Self-Compassion Beneficially Impacts Affect Through Reduced Self-Criticism. *Frontiers in Psychology*, *11*. https://doi.org/10.3389/fpsyg.2020.01371

Krieger, T., Altenstein, D., Baettig, I., Doerig, N., & Holtforth, M. G. (2013). Self-Compassion in Depression: Associations With Depressive Symptoms, Rumination, and Avoidance in Depressed Outpatients. *Behavior Therapy*, *44*(3), 501–513. https://doi.org/10.1016/j.beth.2013.04.004

Lefebvre, J., Montani, F., Courcy, F., & Dagenais‐Desmarais, V. (2021). Self‐compassion at work: A key for enhancing well‐being and innovation through social safeness at multiple levels. *Canadian Journal of Administrative Sciences / Revue Canadienne Des Sciences de l’Administration*, *38*(4), 398–413. https://doi.org/10.1002/cjas.1599

Lenferink, L. I. M., Eisma, M. C., de Keijser, J., & Boelen, P. A. (2017). Grief rumination mediates the association between self-compassion and psychopathology in relatives of missing persons. *European Journal of Psychotraumatology*, *8*(sup6). https://doi.org/10.1080/20008198.2017.1378052

Li, A., & Wang, S. (2024). Self-compassion and general well-being of COVID-19 patients: a moderated mediation model of perceived control and positive coping. *Current Psychology*, *43*(13), 11854–11864. https://doi.org/10.1007/s12144-023-05303-8

Li, A., Wang, S., Cai, M., Sun, R., & Liu, X. (2021). Self-compassion and life-satisfaction among Chinese self-quarantined residents during COVID-19 pandemic: A moderated mediation model of positive coping and gender. *Personality and Individual Differences*, *170*, 110457. https://doi.org/10.1016/j.paid.2020.110457

Liu, C., Lin, P., & Xiong, Z. (2024). Self-Compassion and Psychological Flourishing Among College Students: The Mediating Role of Hope and the Moderating Role of Emotion Regulation. *Behavioral Sciences*, *14*(12), 1149. https://doi.org/10.3390/bs14121149

Maddock, A. (2024a). Psychological Protective and Risk Factors for Depression, Anxiety and Mental Well-Being in Social Workers. *The British Journal of Social Work*, *54*(8), 3773–3793. https://doi.org/10.1093/bjsw/bcae116

Maddock, A. (2024b). Testing Mindfulness Mechanisms of Action on the Stress and Burnout of Social Workers. *Mindfulness*, *15*(5), 1149–1161. https://doi.org/10.1007/s12671-024-02353-2

Maddock, A. (2025). Examining Potential Psychological Protective and Risk Factors for Stress and Burnout in Social Workers. *Clinical Social Work Journal*, *53*(2), 140–155. https://doi.org/10.1007/s10615-024-00924-3

Maddock, A., Hevey, D., D’Alton, P., & Kirby, B. (2020). Examining Individual Differences in Wellbeing, Anxiety and Depression in Psoriasis Using a Clinically Modified Buddhist Psychological Model. *Journal of Clinical Psychology in Medical Settings*, *27*(4), 842–858. https://doi.org/10.1007/s10880-019-09686-4

Makadi, E., & Koszycki, D. (2020). Exploring Connections Between Self-Compassion, Mindfulness, and Social Anxiety. *Mindfulness*, *11*(2), 480–492. https://doi.org/10.1007/s12671-019-01270-z

McBride, N. L., Bates, G. W., Elphinstone, B., & Whitehead, R. (2022). Self‐compassion and social anxiety: The mediating effect of emotion regulation strategies and the influence of depressed mood. *Psychology and Psychotherapy: Theory, Research and Practice*, *95*(4), 1036–1055. https://doi.org/10.1111/papt.12417

Min, L., Jianchao, N., & Mengyuan, L. (2022). The influence of self-compassion on mental health of postgraduates: Mediating role of help-seeking behavior. *Frontiers in Psychology*, *13*. https://doi.org/10.3389/fpsyg.2022.915190

Morgenroth, O., Petersen, L., Frey, N., & Reinecke, A. (2022). The predictive value of self‐compassion for psychological adjustment in left ventricular assist device patients: an observational study. *ESC Heart Failure*, *9*(4), 2378–2387. https://doi.org/10.1002/ehf2.13931

Mosewich, A. D., Sabiston, C. M., Kowalski, K. C., Gaudreau, P., & Crocker, P. R. E. (2019). Self-Compassion in the Stress Process in Women Athletes. *The Sport Psychologist*, *33*(1), 23–34. https://doi.org/10.1123/tsp.2017-0094

Munroe, M., Al-Refae, M., Chan, H. W., & Ferrari, M. (2022). Using self-compassion to grow in the face of trauma: The role of positive reframing and problem-focused coping strategies. *Psychological Trauma: Theory, Research, Practice, and Policy*, *14*(S1), S157–S164. https://doi.org/10.1037/tra0001164

Murfield, J., Moyle, W., O’Donovan, A., & Ware, R. S. (2020). The Role of Self-Compassion, Dispositional Mindfulness, and Emotion Regulation in the Psychological Health of Family Carers of Older Adults. *Clinical Gerontologist*, 1–13. https://doi.org/10.1080/07317115.2020.1846650

Nguyen, T. M., & Le, G. N. H. (2021). The influence of COVID-19 stress on psychological well-being among Vietnamese adults: The role of self-compassion and gratitude. *Traumatology*, *27*(1), 86–97. https://doi.org/10.1037/trm0000295

Norman, D., Correia, H., & Paulik, G. (2020). An exploration of relationship between self‐compassion and voice‐related distress in people who hear voices. *Journal of Clinical Psychology*, *76*(10), 1984–1994. https://doi.org/10.1002/jclp.22975

Ozonder Unal, I., & Ordu, C. (2023). Alexithymia, Self-Compassion, Emotional Resilience, and Cognitive Emotion Regulation: Charting the Emotional Journey of Cancer Patients. *Current Oncology*, *30*(10), 8872–8887. https://doi.org/10.3390/curroncol30100641

Özönder Ünal, I., Ünal, C., Duymaz, T., & Ordu, C. (2023). The relationship between psychological flexibility, self-compassion, and posttraumatic growth in cancer patients in the COVID-19 pandemic. *Supportive Care in Cancer*, *31*(7), 428. https://doi.org/10.1007/s00520-023-07891-4

Peng, Y., & Ishak, Z. (2024). The role of emotion regulation strategies as the mediator between self-compassion and depression among undergraduates in Yunnan province, China. *Discover Mental Health*, *4*(1), 56. https://doi.org/10.1007/s44192-024-00114-0

Pérez-Aranda, A., García-Campayo, J., Gude, F., Luciano, J. V., Feliu-Soler, A., González-Quintela, A., López-del-Hoyo, Y., & Montero-Marin, J. (2021). Impact of mindfulness and self-compassion on anxiety and depression: The mediating role of resilience. *International Journal of Clinical and Health Psychology*, *21*(2), 100229. https://doi.org/10.1016/j.ijchp.2021.100229

Phillips, W. J. (2018). Future-outlook mediates the association between self-compassion and well-being. *Personality and Individual Differences*, *135*, 143–148. https://doi.org/10.1016/j.paid.2018.07.006

Pyszkowska, A., Åström, E., & Rönnlund, M. (2024). Deviations from the balanced time perspective, cognitive fusion, and self-compassion in individuals with or without a depression diagnosis: different mean profiles but common links to depressive symptoms. *Frontiers in Psychology*, *14*. https://doi.org/10.3389/fpsyg.2023.1290676

Pyszkowska, A., & Rönnlund, M. (2021). Psychological Flexibility and Self-Compassion as Predictors of Well-Being: Mediating Role of a Balanced Time Perspective. *Frontiers in Psychology*, *12*. https://doi.org/10.3389/fpsyg.2021.671746

Quang, A. M. T., Van Pham, M., Mai, T. T., Le, G. N. H., & Song, G. A. N. (2022). Self-compassion and Students’ Well-Being Among Vietnamese Students: Chain Mediation Effect of Narcissism and Anxiety. *Journal of Rational-Emotive & Cognitive-Behavior Therapy*, *40*(3), 618–633. https://doi.org/10.1007/s10942-021-00431-1

Raes, F. (2010). Rumination and worry as mediators of the relationship between self-compassion and depression and anxiety. *Personality and Individual Differences*, *48*(6), 757–761. https://doi.org/10.1016/j.paid.2010.01.023

Rakhimov, A., Ong, J., Realo, A., & Tang, N. K. Y. (2023). Being kind to self is being kind to sleep? A structural equation modelling approach evaluating the direct and indirect associations of self-compassion with sleep quality, emotional distress and mental well-being. *Current Psychology*, *42*(16), 14092–14105. https://doi.org/10.1007/s12144-021-02661-z

Rehman, S., Addas, A., Rehman, E., & Khan, M. (2024). The Mediating Roles of Self-Compassion and Emotion Regulation in the Relationship Between Psychological Resilience and Mental Health Among College Teachers. *Psychology Research and Behavior Management*, *Volume 17*, 4119–4133. https://doi.org/10.2147/PRBM.S491822

Ristvej, A. J., McLaren, S., & Goldie, P. D. (2024). The Relations Between Self-Warmth, Self-Coldness, Internalized Heterosexism, and Depressive Symptoms Among Sexual Minority Men: A Moderated-Mediation Model. *Journal of Homosexuality*, *71*(10), 2478–2502. https://doi.org/10.1080/00918369.2023.2245523

Roxas, M. M., David, A. P., & Aruta, J. J. B. R. (2019). Compassion, Forgiveness and Subjective Well-Being among Filipino Counseling Professionals. *International Journal for the Advancement of Counselling*, *41*(2), 272–283. https://doi.org/10.1007/s10447-019-09374-w

Salehi, N., Afrashteh, M. Y., Majzoobi, M. R., Ziapour, A., Janjani, P., & Karami, S. (2023). Does coping with pain help the elderly with cardiovascular disease? The association of sense of coherence, spiritual well-being and self-compassion with quality of life through the mediating role of pain self-efficacy. *BMC Geriatrics*, *23*(1), 393. https://doi.org/10.1186/s12877-023-04083-x

Shi, X., Zhang, W., Chen, X., & Zhu, Y. (2025). Longitudinal Relations among Self-Compassion, Self-Esteem, and Depressive Symptoms in College Students: Disentangling the Within-Person Process from Stable Between-Person Differences. *Journal of Youth and Adolescence*, *54*(1), 255–270. https://doi.org/10.1007/s10964-024-02069-5

Shin, J. Y. (2019). “Will I find a job when I graduate?”: Employment anxiety, self-compassion, and life satisfaction among South Korean college students. *International Journal for Educational and Vocational Guidance*, *19*(2), 239–256. https://doi.org/10.1007/s10775-018-9378-1

Skinner, C. M., & Kuijer, R. G. (2024). Self-compassion and health-related quality of life in individuals with endometriosis. *Psychology & Health*, 1–18. https://doi.org/10.1080/08870446.2024.2325506

Sperandio, K. R., Gutierrez, D., Kirk, M., Lopez, J., & Nathaniel Mason, W. (2022). Post-Traumatic Growth After the Drug-Related Death of a Loved One: Understanding the Influence of Self-Compassion and Hope. *The Family Journal*, *30*(3), 390–400. https://doi.org/10.1177/10664807211052479

Stephenson, E., Watson, P. J., Chen, Z. J., & Morris, R. J. (2018). Self-Compassion, Self-Esteem, and Irrational Beliefs. *Current Psychology*, *37*(4), 809–815. https://doi.org/10.1007/s12144-017-9563-2

Townshend, K., & Caltabiano, N. (2019). Self-Compassion and Mindfulness: Modeling Change Processes Associated with the Reduction of Perinatal Depression. *Journal of Child and Family Studies*, *28*(7), 1790–1802. https://doi.org/10.1007/s10826-019-01371-2

Tran, M. A. Q., Khoury, B., Chau, N. N. T., Van Pham, M., Dang, A. T. N., Ngo, T. V., Ngo, T. T., Truong, T. M., & Le Dao, A. K. (2024). The Role of Self-Compassion on Psychological Well-Being and Life Satisfaction of Vietnamese Undergraduate Students During the COVID-19 Pandemic: Hope as a Mediator. *Journal of Rational-Emotive & Cognitive-Behavior Therapy*, *42*(1), 35–53. https://doi.org/10.1007/s10942-022-00487-7

Ueno, Y., & Amemiya, R. (2024). Mediating Effects of Resilience Between Mindfulness, Self-compassion, and Psychological Distress in a Longitudinal Study. *Journal of Rational-Emotive & Cognitive-Behavior Therapy*, *42*(4), 896–906. https://doi.org/10.1007/s10942-024-00553-2

Vidal, J., Ciudad-Fernández, V., Navarrete, J., Soler, J., Schmidt, C., Molinari, G., & Cebolla, A. (2024). From self-criticism to self-compassion: exploring the mediating role of two emotion dysregulation variables in their relationship to depressive symptoms. *Current Psychology*, *43*(32), 26539–26547. https://doi.org/10.1007/s12144-024-06325-6

Voon, S. P., Lau, P. L., Leong, K. E., & Jaafar, J. L. S. (2022). Self-Compassion and Psychological Well-Being Among Malaysian Counselors: The Mediating Role of Resilience. *The Asia-Pacific Education Researcher*, *31*(4), 475–488. https://doi.org/10.1007/s40299-021-00590-w

Wadsworth, L. P., Forgeard, M., Hsu, K. J., Kertz, S., Treadway, M., & Björgvinsson, T. (2018). Examining the Role of Repetitive Negative Thinking in Relations Between Positive and Negative Aspects of Self-compassion and Symptom Improvement During Intensive Treatment. *Cognitive Therapy and Research*, *42*(3), 236–249. https://doi.org/10.1007/s10608-017-9887-0

Wang, Y., Fu, T., Wang, J., Chen, S., & Sun, G. (2024). The relationship between self-compassion, coping style, sleep quality, and depression among college students. *Frontiers in Psychology*, *15*. https://doi.org/10.3389/fpsyg.2024.1378181

Wong, C. C. Y., & Yeung, N. C. Y. (2017). Self-compassion and Posttraumatic Growth: Cognitive Processes as Mediators. *Mindfulness*, *8*(4), 1078–1087. https://doi.org/10.1007/s12671-017-0683-4

Wu, D., Ye, B., Tang, C., Xue, J., Yang, Q., & Xia, F. (2022). Self-Compassion and Authentic-Durable Happiness During COVID-19 Pandemic: The Mediating Role of Meaning of Life and the Moderating Role of COVID-19 Burnout. *Psychology Research and Behavior Management*, *Volume 15*, 3243–3255. https://doi.org/10.2147/PRBM.S380874

Xie, Q. (2023). Are Mindfulness and Self-Compassion Related to Peace of Mind? The Mediating Role of Nonattachment. *Psychological Reports*. https://doi.org/10.1177/00332941231198511

Xie, Q., Manova, V., & Khoury, B. (2023). How do dispositional mindfulness and self-compassion alleviate loneliness? The mediating role of rejection sensitivity. *Current Psychology*, *42*(27), 23712–23721. https://doi.org/10.1007/s12144-022-03549-2

Xu, L., Shi, J., & Li, C. (2024). Addressing psychosomatic symptom distress with mindfulness-based cognitive therapy in somatic symptom disorder: mediating effects of self-compassion and alexithymia. *Frontiers in Psychiatry*, *15*. https://doi.org/10.3389/fpsyt.2024.1289872

Yang, Y., Zhang, M., & Kou, Y. (2016). Self-compassion and life satisfaction: The mediating role of hope. *Personality and Individual Differences*, *98*, 91–95. https://doi.org/10.1016/j.paid.2016.03.086

Yela, J. R., Crego, A., Buz, J., Sánchez‐Zaballos, E., & Gómez‐Martínez, M. Á. (2022). Reductions in experiential avoidance explain changes in anxiety, depression and well‐being after a mindfulness and self‐compassion (MSC) training. *Psychology and Psychotherapy: Theory, Research and Practice*, *95*(2), 402–422. https://doi.org/10.1111/papt.12375

Ying, Y.-W. (2009). CONTRIBUTION OF SELF-COMPASSION TO COMPETENCE AND MENTAL HEALTH IN SOCIAL WORK STUDENTS. *Journal of Social Work Education*, *45*(2), 309–323. https://doi.org/10.5175/JSWE.2009.200700072

Zeng, P., Nie, J., Geng, J., Wang, H., Chu, X., Qi, L., Wang, P., & Lei, L. (2023). Self‐compassion and subjective well‐being: A moderated mediation model of online prosocial behavior and gratitude. *Psychology in the Schools*, *60*(6), 2041–2057. https://doi.org/10.1002/pits.22849

Zerach, G. (2025). The beneficial effect of self-compassion on PTSD and complex PTSD symptoms among Israeli female veterans: The role of coping strategies. *Psychology of Consciousness: Theory, Research, and Practice*, *12*(2), 253–268. https://doi.org/10.1037/cns0000409

Zhang, W., Chen, X., Zhu, Y., & Shi, X. (2025). Within-Person Relationships Among Self-Compassion, Emotion Regulation Difficulties, and Anxiety Symptoms: Testing an Emotion Regulation Model of Self-Compassion. *Journal of Youth and Adolescence*, *54*(5), 1163–1177. https://doi.org/10.1007/s10964-024-02122-3

Zhao, F.-F., Yang, L., Ma, J.-P., & Qin, Z.-J. (2022). Path analysis of the association between self-compassion and depressive symptoms among nursing and medical students: a cross-sectional survey. *BMC Nursing*, *21*(1), 67. https://doi.org/10.1186/s12912-022-00835-z

Zhou, L., Chen, J., Liu, X., Lu, D., & Su, L. (2013). Negative Cognitive Style as a Mediator Between Self-Compassion and Hopelessness Depression. *Social Behavior and Personality: An International Journal*, *41*(9), 1511–1518. https://doi.org/10.2224/sbp.2013.41.9.1511

Zhu, L., Wang, J., Liu, S., Xie, H., Hu, Y., Yao, J., Ranchor, A. V., Schroevers, M. J., & Fleer, J. (2020). Self-Compassion and Symptoms of Depression and Anxiety in Chinese Cancer Patients: the Mediating Role of Illness Perceptions. *Mindfulness*, *11*(10), 2386–2396. https://doi.org/10.1007/s12671-020-01455-x

Zhu, L., Wei, L., Xiaomin, Y., Zhao, J., Yu, Y., Sun, S., Wang, X., Yao, J., & Xie, J. (2022). Self‐compassion and fear of cancer recurrence in Chinese breast cancer patients: The mediating role of maladaptive cognitive styles. *Psycho-Oncology*, *31*(12), 2185–2192. https://doi.org/10.1002/pon.6070

Zipagan, F. B., & Galvez Tan, L. J. T. (2023). From Self-Compassion to Life Satisfaction: Examining the Mediating Effects of Self-Acceptance and Meaning in Life. *Mindfulness*, *14*(9), 2145–2154. https://doi.org/10.1007/s12671-023-02183-8
